# Evaluating the use of artificial intelligence and human guidance to teach generic and specialist skills in breaking bad news: A mixed methods exploration

**DOI:** 10.1016/j.clinme.2026.100649

**Published:** 2026-09-28

**Authors:** Miriam E. Armstrong, Philip Bright, Stephen Glen, David Marshall, Michael C. Jones

**Affiliations:** a Federation of the Royal Colleges of Physicians of the United Kingdom, 11 St Andrews Place, Regent’s Park, London NW1 4LE, United Kingdom; b NHS England West Midlands Office, 23 Stephenson Street, Birmingham B2 4HQ, United Kingdom

**Keywords:** Artificial intelligence, Breaking bad news, Postgraduate training, Mixed methods: Qualitative analysis

## Abstract

**Objective:**

Breaking bad news (BBN) is specified in the General Medical Council’s Generic Professional Capabilities framework, which sets out core skills to be developed alongside curricula-specified capabilities in postgraduate training. To date, a wide variety of approaches to teaching BBN have been used, however, the application of artificial intelligence (AI) to learning in this field remains in its early stages and requires further investigation.

**Design, setting, participants and intervention:**

Two approaches were compared in response to the question *What is the best way to break bad news?: a* human-facilitated discussion versus an AI-generated response using ChatGPT-4. Both approaches produced high-level guidance condensed into short statements for online mixed methods evaluation by experienced medical educators.

**Outcome measures and results:**

41 doctors from 17 specialties and eight lay representatives provided free-text commentary as part of the assessment. Overall findings were compared with popular patient-focused BBN protocols, such as SPIKES, with qualitative analysis providing more details of skills requirements for generic and specialist levels of practice by adding more depth and nuance via three themes: patient experience, professionalism and access to support. Both approaches matched well with patient-focused protocols albeit they also addressed doctors’ debriefing after BBN.

**Conclusions:**

Overall, AI produced more comprehensive, highly rated guidance suitable for early careers, but qualitative evaluation highlighted the need for more specialised skills, their nature and application, plus differences in preferred style and content between approaches. The value of tailoring communication, via a personalised approach developed from real-life experience, was also confirmed.

## Highlights

• AI-produced guidance aligns well with popular patient protocols, such as SPIKES.
• AI uses clear language that supports stepwise approach suitable for early training.
• Experience helps develop a unique individual, instead of a ‘tick box’, approach.
• Priorities for different approaches vary with specialty and circumstance.
• Combined AI and human experience can optimise development of specialist BBN skills.

## Graphical abstract

**Figure:**
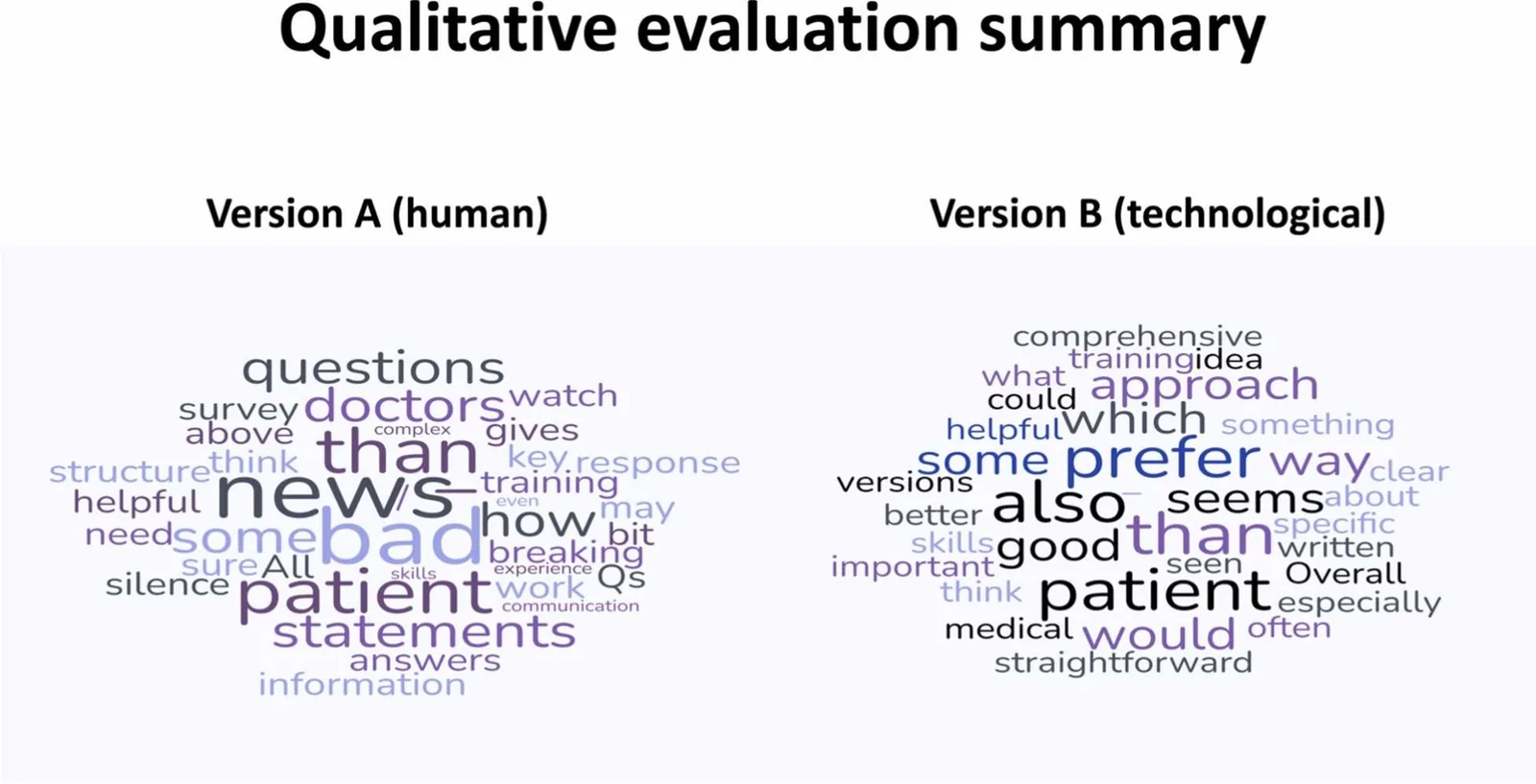

## Introduction

The effective teaching of BBN has been well-studied.^1^, ^2^, ^3^, ^4^, ^5^ A variety of approaches from lectures, simulation,^6^, ^7^ workshops,^8^, ^9^, ^10^ role play,^11^ group discussion and reflective practice have been trialled, often with structured communications frameworks incorporated, for example the SPIKES^12^ (Setting up; Perception; Invitation; Knowledge; Emotions; Strategy/Summarise) or BREAKS^13^ (Background; Rapport; Explore; Announce; Kindling; Summarise) mnemonics developed for use in oncology settings. Other variations have been developed for more generalist application, such as the ABCDE^14^ approach (Advance preparation; Building therapeutic environment; Communicate well; Deal with reactions; Encourage emotions). Reviews of available evidence indicate that more formalised training, including structured protocols,^15^ deliver better outcomes such as improved confidence and skills in news delivery^2^ plus more effective preparation for clinical examinations, such as the MRCP(UK) PACES.^16^

Given the fundamental importance of BBN to medical practice, teaching typically starts at undergraduate level^17^ but is recommended to continue throughout postgraduate education and indeed the career span.^18^, ^19^ At a postgraduate level, BBN is a core requirement of the General Medical Council’s (GMC) Generic Professional Capabilities (GPCs) framework^20^ that, from May 2017, has underpinned all UK postgraduate curricula and defined the core knowledge, skills and behaviours that need to be developed alongside meeting specialty-specific curricula requirements:

ʻ*Due to the complex nature of medical practice, doctors in training must develop high levels of communication and interpersonal skills …. They must do this*:

• *with patients, relatives, carers, guardians and others by:*
  • …. communicating effectively and sensitively when breaking bad news and being well prepared to give clear information.’

*(p11, Domain 2 – Professional skills: Communication and interpersonal skills. Generic professional capabilities framework.)*

As a result, individual curricula that used to specify BBN as a core competency, such as internal medicine (IM)^21^ and medical oncology,^22^ now refer to the GPC framework:

ʻ*From May 2017, all postgraduate curricula should be based on higher level learning outcomes and must incorporate the generic professional capabilities. A fundamental component of the GPCs is ensuring that the patient is at the centre of any consultation and decision making …. Trainees are encouraged to reflect on their communication skills throughout every stage of their training.*’

*(p6, JRCPTB IM curriculum)*

The Joint Royal Colleges of Physicians Training Board (JRCPTB) is a UK-wide organisation responsible for setting standards in 30 postgraduate medical curricula on behalf of the Royal Colleges of Physicians of London, Edinburgh and Glasgow. The 30 specialties are classified as either Group 1, which contribute more directly to acute and general medical care, or Group 2 (see supplementary material for more details). The JRCPTB has been exploring alternative ways of teaching and assessing GPCs as part of its quality improvement programme. Some of this work has involved facilitated discussions between trainers and resident doctors around BBN, to encourage learning by collective reflection on others’ experiences. As part of this wider programme, the JRCPTB wished to evaluate if AI might further augment this work and if any benefits applied equally to Group 1 and 2 specialties?

## Methods

Guidance on teaching BBN in a medical training environment was developed in the form of high-level principles via a traditional (human-facilitated) and a technological (paid-for ChatGPT-4) approach in response to the question ‘What is the best way to break bad news?’. A total of 14 and 19 high-level principles were produced by the human and AI approaches respectively, of which 11 were common to both. All principles were subsequently transformed into a series of short, similarly formatted, online statements to which members of the JRCPTB postgraduate specialty training committees (blind to the method of production) were invited to compare and rate (using a five-point Likert scale, ranging from strongly agree to strongly disagree) and to provide feedback on each statement via a free text option (see supplementary material for the online consultation tool). Additional details of the quantitative assessment design and limitations have been published elsewhere.^23^ The purpose of the qualitative assessment was to explore:

• Qualitative differences between the human and AI-generated ‘guidance’
• If significant differences between Group 1 and 2 specialty responses existed
• What might be the optimal learning approach to develop BBN skills?

Braun and Clarke’s approach to thematic analysis was adopted.^24^ This is an iterative process where exploration and familiarity with unprocessed data leads to the evolution of primary themes and secondary sub-themes for analysis. One author (MA) undertook the initial organisation of data as per Box 1, with an independent review conducted by a second author (MCJ), which resulted in a coalescing of identified sub-themes, for example, BBN skills specified by lay representatives were removed from Patient Experience and subsumed into longer-term Skills Development.

Box 1 **Primary qualitative themes and sub-themes with rationale.**

**Table:** 

Primary theme and rationale	Sub-themes with rationale and examples
**Patient experience** – matters viewed from patients’ or relatives’ perspectives	Improving patient experience – e.g. tailoring information, pausing to check understanding
**Professionalism** – focus on optimal approach or specific learning considerations to improve doctors’ capabilities.	Workplace-based practice improvements – e.g. preparation for consultation Workplace-related soft skills development – e.g. building rapport or relationships Dealing with uncertainty or difficulty – e.g. managing uncertainty or difficulty well
**Access to support and further training** – guidance available to doctors to enhance learning or wellbeing.	Near-event reflection/debrief/wellbeing – e.g. debriefing with colleagues Longer-term skills development – e.g. building on experience. Training or study methods – e.g. language awareness, allowing for ambiguity

Free-text comments were mapped to a framework plotting Group 1 and Group 2 specialty responses plus lay representatives’ data against human- and AI-generated BBN statements (details provided as supplementary material). All meaningful comments were coded and grouped into themes and sub-themes as described in Box 1, which were then explored for commonalities and differences within the framework.

## Results

41 doctors from 17 specialties and eight lay representatives provided free-text commentary, representing 61% of doctors (68% Group 1; 50% Group 2 specialties) and 67% of lay representatives taking part in the overall assessment. The largest groups of respondents were lay representatives (n = 8), Medical Oncology (n = 8) and Palliative Care (n = 7) with representation across the remaining specialties being evenly spread (see supplementary material for more details).

Results from the quantitative assessment were used to inform the exploration of qualitative findings. For example, particular attention was focused where the largest disagreement existed between the human- and AI-generated ‘guidance’ and Group 1 and 2 specialties as opposed to areas of high agreement, although these were also explored.

Table 1 provides an overview of qualitative and quantitative findings mapped to commonly used patient-focused BBN protocols, such as SPIKES, to create a broader context for the discussion of qualitative themes. Fig. 1 provides an overview of the differences between Group 1 and 2 specialty responses for a similar purpose.

**Table 1:** Common BBN statements with percentage respondent agreement and qualitative themes and sub-themes, mapped to commonly used patient-focused protocols.

	BBN statements common to both approaches: Areas of similarity (paraphrased for brevity) with mapping to core domains of patient-focused BBN protocols	Traditional (human) approach % agreement (N = 80) *Qualitative themes/sub-themes (n = frequency of theme in responses)*	Technological (AI) approach % agreement (N = 75) *Qualitative themes/sub-themes (n = frequency of theme in responses)*
1	**Prior to the discussion:** Understand basics, such as prognosis and who is involved in care, before talking to patient. SPIKES: Setting up BREAKS: Background ABCDE: Advance preparation	91	96
		*Patient experience (n = 12)* *Workplace practice (n = 9)* *Training methods (n = 1)*	*Patient experience (n = 12)* *Workplace practice (n = 2)* *Dealing with uncertainty (n = 4)*
2	**Find the right location:** Private and quiet environment is essential. SPIKES: Setting up BREAKS: Background ABCDE: Advance preparation	99	100
		*Patient experience (n = 12)* *Workplace practice (n = 11)* *Training methods (n = 1)*	*Patient experience (n = 3)* *Workplace practice (n = 10)* *Training methods (n = 1)*
3	**Emotional preparation:** Accept seriousness of situation and need to maintain composure throughout. SPIKES: Setting up BREAKS: Background ABCDE: Advance preparation	99	99
		*Patient experience (n = 1)* *Workplace practice (n = 1)* *Workplace soft skills (n = 9)* *Reflection/debriefing (n = 1)* *Skills development (n = 1)* *Training methods (n = 3)*	*Workplace practice (n = 4)* *Workplace soft skills (n = 2)*
4	**Assess engagement:** Tailor communication to their knowledge and understanding. SPIKES: Perception, Invitation BREAKS: Rapport, Explore ABCDE: Build therapeutic environment	91	96
		*Patient experience (n = 12)* *Workplace practice (n = 14)* *Training methods (n = 1)*	*Patient experience (n = 8)* *Workplace practice (n = 8)* *Workplace soft skills (n = 1)* *Training methods (n = 2)*
5	**Respect patient preferences:** Be guided by the patient’s preferences to receive information. SPIKES: Perception, Invitation, Knowledge BREAKS: Rapport, Explore ABCDE: Communicate well	91	93
		*Patient experience (n = 5)* *Workplace practice (n = 5)* *Workplace soft skills (n = 1)* *Reflection/debriefing (n = 3)* *Training methods (n = 4)*	*Patient experience (n = 9)* *Workplace practice (n = 10)* *Workplace soft skills (n = 2)* *Training methods (n = 2)*
6	**Show compassion:** Show some humanity. Clearly state the bad news but do so in a compassionate tone. SPIKES: Emotions with empathy, Summarise BREAKS: Announce, Kindle, Summarise ABCDE: Communicate well	98	100
		*Workplace practice (n = 1)* *Workplace soft skills (n = 1)* *Skills development (n = 2)* *Training methods (n = 2)*	*Patient experience (n = 3)* *Workplace practice (n = 3)* *Training methods (n = 2)*
7	**Allow for emotions:** Acknowledge and validate the patient’s or family’s emotions. SPIKES: Emotions with empathy BREAKS: Announce, Kindle ABCDE: Deal with reactions, Encourage emotions	88	96
		*Workplace practice (n = 1)* *Workplace soft skills (n = 3)* *Dealing with uncertainty (n = 7)* *Reflection/debriefing (n = 5)* *Skills development (n = 1)* *Training methods (n = 2)*	*Patient experience (n = 2)* *Workplace soft skills (n = 6)* *Reflection/debriefing (n = 1)* *Training methods (n = 4)*
8	**Take time to manage own response:** Manage your own emotional response before moving to next patient. SPIKES, BREAKS, ABCDE: Not relevant due to focus on managing doctors’ responses	89	100
		*Workplace practice (n = 5)* *Dealing with uncertainty (n = 2)* *Reflection/debriefing (n = 3)*	*Dealing with uncertainty (n = 1)* *Reflection/debriefing (n = 3)*
9	**Reflect on the experience:** Consider what went well, what could be improved and own emotional impact. SPIKES, BREAKS, ABCDE: Not relevant due to focus on managing doctors’ responses	96	96
		*Dealing with uncertainty (n = 3)* *Reflection/debriefing (n = 4)*	*Workplace practice (n = 1)* *Reflection/debriefing (n = 3)*
10	**Seek feedback:** Seek feedback from peers or more experienced colleagues to improve skills. SPIKES, BREAKS, ABCDE: Not relevant due to focus on managing doctors’ responses	95	95
		*Workplace practice (n = 1)* *Reflection/debriefing (n = 2)*	*Reflection/debriefing (n = 3)*
11	**Emotional debriefing:** Allow time to process emotional impact and gain insights for future scenarios. SPIKES, BREAKS, ABCDE: Not relevant due to focus on managing doctors’ responses	90	96
		*Workplace soft skills (n = 7)* *Reflection/debriefing (n = 9)* *Training methods (n = 5)*	*Workplace soft skills (n = 2)* *Reflection/debriefing (n = 2)*

**Fig. 1:**
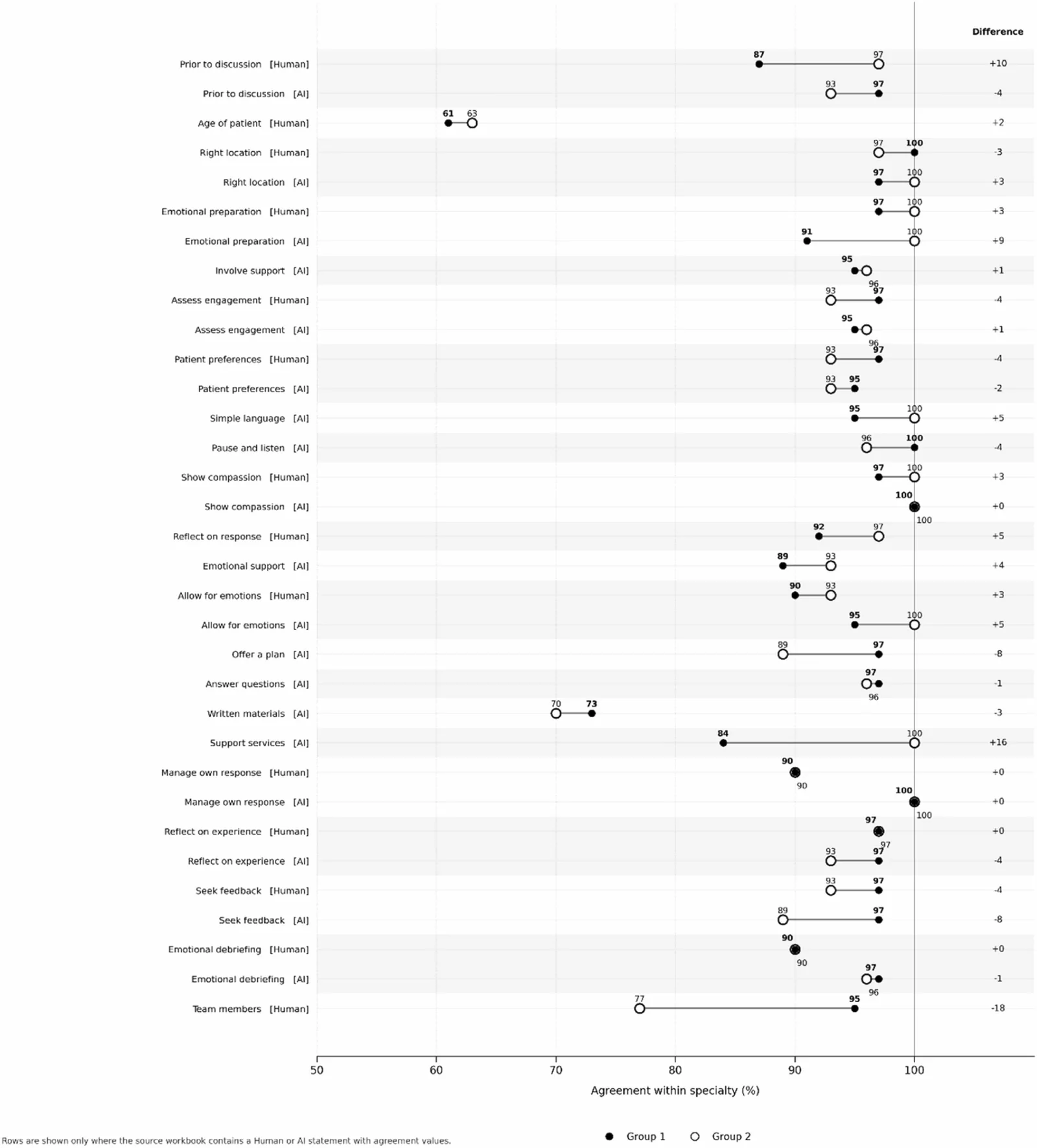
Human versus AI-generated BBN statements by Group 1 and 2 specialty agreement.

Major discussion points are presented by qualitative themes and sub-themes in Table 2. The most frequently commented on sub-themes were workplace practice (n = 163), patient experience (n = 113) and training methods (n = 90). All other sub-themes were much less commented on (workplace soft skills, n = 56; reflection, n = 42; dealing with uncertainty, n = 34; skills development, n = 27) albeit frequency reflected the number of topics stimulating interest, for example prompting clarification or a qualification to the statement, rather than an indication of agreement or disagreement (which was measured quantitatively, as per Table 1).

**Table 2:** Qualitative themes with quotes illustrative of main discussion points.

Qualitative sub-theme	Sub-theme frequency (n)	Quotes illustrative of points of interest within each theme or sub-theme
***Patient experience***		
Improving patient experience	Human (n = 60)	*Prior to the discussion* Group 1: I think essential to try and gain some kind of rapport (i.e. who's with them, anyone important they want with them, how they are feeling etc). (Palliative Medicine) Group 2: Very important to try and establish a rapport. (Medical Oncology) Lay representative: Person centred care needs to apply and taking that time is an indication of personal care and interest in the individual. *Age of the patient* Group 1: This is person dependent, not age dependent. (Palliative Medicine) Group 2: Although this is a very broad statement and doesn't mean that elderly patients are not upset to be told of a terminal diagnosis. (Medical Oncology) Lay representative: Age ‘may’ be an important consideration.
	AI (n = 53)	*Respect patient preferences* Group 1: This is often a process – not just one conversation. (Geriatric Medicine) Group 2: Always tricky to ask how much patients want to know, this is often something that is gauged by previous interactions with patient. (Audiovestibular Medicine) Lay representative: Sometimes you break bad news, and the patient does not understand it.
***Professionalism***		
Workplace-based practice improvements	Human (n = 62)	*Assess engagement* Group 1: Often, cancer patients receive lots of news regarding an active treatment plan but not much about what happens should they deteriorate. (Geriatric Medicine) Group 2: Too often I hear cancer cured. (Pharmaceutical Medicine) Lay representative: You need to be sure there is no hope – because hope is an important bit of kit that can help patients manage their wellbeing and quality of life.
	AI (n = 101)	*Assess engagement* Group 1: Sometimes this can come across as patronising for some patients depending how it is phrased as well. (Palliative Medicine) Group 2: Not always necessary – and can irritate some patients when they've already answered questions with other staff, although a degree of this is always important. (Medical Oncology) Lay representative: Caveat is that this may come across as a bit of an interrogation! *Offer written materials* Group 1: Written information that includes relevant contact numbers very important. If there is lot of information to process then may be better to arrange a further discussion soon, when the additional information is shared and discussed. (Respiratory Medicine) Group 1: Realistically not always available for patients’ individual situations – yes if available and directly relevant/validated. (Rheumatology) Group 2: Make sure you check the content of written information – patients will take it as gospel. (Haematology) Group 2: This isn't always helpful and some people don't want the confirmation of the bad news in writing. However, if this is the case, the information conveyed should be recorded contemporaneously in the patient's notes. (Clinical Genetics) Lay representative: Do it but don't force it. *Refer to support services* Group 1: I think offering support services too quickly can also imply grief is an abnormal reaction when actually it is normal to feel upset etc. I think offering these when people are having pathological responses after allowing the information to settle in is better. (Palliative Medicine) Group 2: Needs to be done in a way that patients don't feel they are being fobbed off onto another service. (Audiovestibular Medicine)
Workplace-related soft skills development	Human (n = 31)	*Emotional preparation* Group 1: Empathy is critical but the manner in which this is expressed will need to be balanced and specific to circumstances. One size does not fit all. (Respiratory Medicine) Group 2: Empathy isn’t just body language or words, it’s about listening, hearing and making the patient feel included and that their views matter. (Paediatric Cardiology)
	AI (n = 25)	*Pause and listen* Group 1: Active listening is a real skill. (Geriatric Medicine) Group 2: Not rushing through a prepared spiel is important, listening to reaction will help guide further approach. (Medical Oncology) *Allow for emotions* Group 1: Commentating on the emotions of the patient shouldn't always be volunteered. In my experience it is irritating for some patients and their families, particularly when they are surprised by the information. (Respiratory Medicine) Group 2: Adding specific examples might be taken out of context by less experience and then could be more inflammatory in a challenging consultation, especially ʻI can see this is very upsetting’. (Medical Oncology)
Dealing with uncertainty or difficulty	Human (n = 25)	*Reflect on the response* Group 1: I have had some awful interactions centred around you are giving up etc. Need for support for the breaker of the bad news is important. (Geriatric Medicine) Group 2: This is a sign of maturity or lack of it as a doctor, the more experienced you become the less the patient impact is on you personally - it is our / your job and we are trained. (Medical Oncology) Lay representative: Emphasis is focusing on the patient - they should be given latitude, and it might seem wrong if you are tempted to feel a victim following the situation.
	AI (n = 9)	*Prior to discussion* Group 1: Uncertainty is often a part of these conversations and being honest and open about this is important. (Geriatric Medicine) Group 2: Ideal but not always possible especially if in situation where ʻcaught out’ in giving bad news that was not expected within the consultation. (Medical Oncology) *Answer questions* Group 1: Expressing uncertainty is ok as well. (Rheumatology) Group 2: Unfortunately, uncertainty is unavoidable at some points for us. (Medical Oncology)
***Access to support and further training***		
Near-event reflection/debrief/wellbeing	Human (n = 31)	*Allow for emotions* Group 1: Reflecting why patients or families may be aggressive or critical is important and can be a response to stress/distress. However, it can also be when the communicating doctor is unprepared or doesn't leave a clear plan. I would add something in about the range of reactions patients and families may express and recognise the factors that might impact responses. (Palliative Medicine) Group 2: Important trainees explore this and develop appropriate critical thinking as to the possible rationales, self-reflection on breaking the news technique, and their subsequent resilience and coping strategies. (Audiovestibular Medicine) Lay representative: Difficult situation but not an uncommon reaction. Important medical professionals are taught to manage this both in the situation and afterwards to safeguard themselves. *Manage own response* Group 1: This will depend upon the how the doctor feels and the circumstances. Certainly, shouldn't feel rushed to move on. (Respiratory Medicine) Group 2: Learning to accept you are only human and it will affect you. (Haematology) *Emotional debriefing* Group 1: There is probably a difference between reflection on performance and emotional debrief. Different people are likely to be better at each component (senior colleagues vs friends for instance). (Rheumatology) Group 2: You should be able to approach all seniors on non-clinical issues, if not they have not been trained properly. (Medical Oncology) Lay representative: Needs a 'trustful' setting between all colleagues.
	AI (n = 11)	*Seek feedback* Group 1: For more junior colleagues training in breaking bad news and getting feedback is part of this process. (Geriatric Medicine) Group 2: Feedback from a less experienced colleague can also be valuable. (Haematology)
Longer-term skills development	Human (n = 22)	*Team members* Group 1: This is a core skill of any doctor, whilst it’s important to check beforehand, this shouldn't be a barrier to gaining experience. (Palliative Medicine) Group 2: Breaking bad news is something all doctors should be able to do – it is part of our role. (Medical Oncology) Group 2: Agree and disagree. Depends on the seniority and experience and confidence of the junior staff. I would always check with an IMT doctor particularly if limited oncology experience but would expect my more senior trainees – ST4+ particularly – to be able to break bad news as oncology trainees. (Medical Oncology) Group 2: In general this isn’t something to randomly delegate; how to empower those in more junior positions to decline to do this? (Paediatric Cardiology) Lay representative: This is an expectation of the role and Junior staff need to observe and learn how to do this in a supported way in order to be able to manage this effectively themselves at a later stage.
	AI (n = 5)	There were no illustrative comments identified in this category.
Training or study methods	Human (n = 33)	These comments are explored further in Table 3.
	AI (n = 57)	

Overall, there was broad agreement with the fundamental principles of most statements, albeit with some areas of divergence. For example, there were six statements where disagreement between Group 1 and 2 specialties exceeded five percentage points (Human statements: Team members, 18; Prior to discussion, 10; AI statements: Support services, 16; Emotional preparation, 9; Offer a plan, 8; Seek feedback, 8) with all other areas showing close agreement (Fig. 1), Some of the nuances of these differences are discussed under the major themes below:

### Patient experience

There was considerable agreement in the evaluation of human versus AI plus Group 1, Group 2 specialty and lay statements for patient experience, with comments reflecting the complexity of the discussion and the need for individual adaptations. Patient experience was thought to be enhanced by facilitating good engagement from the outset, including preparation for the discussion and tailoring information to understanding and preferences.

### Professionalism

There was general agreement that sensitivity was critical, emphasising the importance of genuine empathy and active listening whilst avoiding the converse, such as patronising, overly interrogative behaviour or assuming what the patient’s reaction was going to be.

Areas of disagreement included the offer of supplementary services or information, such as counselling or written materials, both for practical reasons such as lack of universal access as well as the timing of the conversation, for example, considering if the patient needed more time to process the information they had already received.

Managing difficulty and uncertainty was regarded as inherent to the task, with doctors expected to both manage their own emotional response and not over-react to displays of negativity or aggression, given the circumstances. The comments revealed some variation in how difficult this might be to achieve in practice, but doctors received training to overcome it.

### Access to support and further training

There was good agreement for reflection or debriefing as a natural follow up to managing challenging situations. Taking time to reflect on difficult experiences and the doctors’ own emotional response was considered important to sustaining long-term practice. The role of colleagues in sharing burdensome scenarios prompted more variation in responses, ranging from separating out work matters from emotional experience to supporting the less experienced, whilst recognising that genuine feedback and reflection is ultimately of developmental benefit.

Comments made directly on the two approaches (human or AI-generated statements) mainly featured in the ‘any other comments’ section at the end of the survey, where the consultation tool also provided the opportunity to provide more general comments comparing the learning style or perceived usefulness of each approach, for example ‘*Please enter any additional thoughts or comments on Version [A or B] here’*. Within this section, respondents offered a critique of the merits of the style of each approach, which were wide-ranging in nature and broadly classified as either positive, neutral or negative (Table 3).

**Table 3:** Free-text comments highlights on the overall merits or disadvantages of each approach.

	Version A (Traditional – human)	Version B (Technological – AI)
Positive	All points above are crucial and important. *[Lay representative]* I prefer the format of Version A as it allows more possibilities for contextualisation. *[Lay representative]* Overall, version A seems more actionable for training. *[Respiratory Medicine]*	Good use of a practical examples of what this might look like. *[Lay representative]* Includes additional information on why something is helpful or recommended *[Geriatric Medicine]* Seems more human and more individualised and focuses on skills rather than specific ʻtasks. It also aligns better with communication skills workshops*. [Palliative Medicine]* I prefer Version B because of the clear language and the good advice given. A is a mixture of giving facts and making suggestions. *[Lay representative]* Overall, I prefer Version B. *[Genitourinary Medicine]* Version B is far better! *[Palliative Medicine]* Version B more comprehensive than version A. *[Cardiologist]* Version B is much clearer and straightforward to answer than Version A. *[Acute Internal Medicine]* Version B is more practical and better suited for a stepwise approach especially while working on a busy medical take. *[Endocrinology and Diabetes Mellitus]* Much prefer version B. Keeps approach as patient centred, gives a clear structure to approach, helpful phrases included that could be used by doctors in training. *[Palliative Medicine]*
Neutral	These statements offer insights and suggestions rather than specific *dos or dont’s* and are a bit more open – although not all are in this format. *[Lay representative]*	As long as it does not sound fake – and is natural for you – use your own words. *[Medical Oncologist]*
Negative	Communication skills are complex and even with experience and training it is not always straight-forward. I think this style of question makes breaking bad news seem more of a tick-box approach. *[Palliative Medicine]* Version A was so poor had it been written to deliberately inflame! *[Geriatric Medicine]*	Longer with more specific examples which I think could lead to misinterpretation or use of these verbatim if seen as ʻbest practice’ which misses the point that each consultation will need slightly different tactics. *[Medical Oncologist]* I did not like B. Too clinical, prescriptive and often not relevant. *[Medical Oncologist]*

Some respondents saw clear value in using elements of both approaches to optimal effect. For example:

• Version B is more factual but less comprehensive and well-rounded than A. *[Lay representative]*
• Elements of both versions are very good. I would suggest combining the parts which are distinct between the two versions. *[Respiratory Medicine]*
• B more straightforward for trainees to read and work through, but loses some of the depth and nuance that was in version A. *[Palliative Medicine]*
• There are things in B which would complement some of those in A and vice versa! *[Paediatric Cardiology]*
• Version A is more general, B is more ʻstep by step’ moment by moment for those with less experience. *[Audiovestibular Medicine]*
  Whilst others highlighted the practical limitations of both approaches. For example:
• I think version B especially (but also A to some extent) creates unrealistic expectations about what can actually be delivered in the NHS. Being able to find 5 min and a quiet room for this important task is usually about all that can be done, the idea that there will regularly be the possibility of written materials or referral to support workers is outlandish outside of specialist services (e.g. in oncology). The idea that nurses or consultants will have sufficient time or motivation to attend is also not realistic. *[Respiratory Medicine]*

Further comments were provided in this section on the overall approach to the task of breaking bad news. For example:

• Focus on clearly understanding the patient and family's view prior to difficult discussions is probably the single most useful thing I was taught in medical school. *[Infectious Diseases and Tropical Medicine]*
• There is no right way to give bad news, the messenger is often killed. Preparation is key, knowledge gives confidence. *[Medical Oncologist]*
• Reassuring as a patient to know this level of thought is going into what might be seen to be really consequential in relieving distress. *[Lay Representative]*

The importance of doctors developing their own personalised approach, tempered by experience and appropriate feedback, was also highlighted. Different specialties tended to emphasise particular aspects of care relevant to their priorities, for example, respiratory and acute medicine focused on more practical aspects, whilst those regularly managing bad news, such as oncology and palliative care, focused on communication nuances.

## Discussion

The online consultation on the ‘best way to break bad news’ produced a wealth of data that collectively confirmed the similarities between the two approaches (human and AI-generated) whilst highlighting the inherent skill and situational complexity required for optimal management.

The richness of the free text data facilitated deeper insight into the merits and weaknesses of each approach. Both matched well with the main components of patient-centred protocols, such as SPIKES, but created additional ‘guidance’ to support doctors debrief after BBN, which lay outside the scope of the patient-centred protocols.

Variation in Group 1 and 2 specialty responses was explored to determine whether training requirements differed between those regularly delivering bad news in acute scenarios from less urgent settings, however, this was not borne out by the findings. Albeit it was expected that the major disciplines regularly involved in breaking bad news (for example, Palliative Medicine, Medical Oncology) would feature strongly in the consultation (having developed a strong interest alongside a high degree of specialist skill) it became apparent that BBN is an important feature for many physician disciplines and the progression of skills from a generic to a specialist level is not only desirable, but essential to patient experience, clinical practice and doctors’ wellbeing.

Overall, the disparities observed from the mixed methods exploration were not significant enough to detect any qualitative differences in content or acceptability of the human or AI-generated statements. Essentially the underpinning principles were similar, whilst the best way to apply this approach in practice was subject to context and interpretation.

The judicious use of AI alongside traditional methods of learning – informed and updated by practical experience with high-quality supervision and reflection – can enhance the development of these skills in the workplace. In particular, that a stepwise process from ‘basic generic skills’ (requiring clear guidance on the main aspects and options for consideration) to a more personalised ‘specialist approach’ incorporating broader experience and preferred communication style or techniques into the framework, should be supported in practice.

The limitations of this study apply to both the qualitative and quantitative aspects, with the latter described in more detail elsewhere.^23^ Essentially there is limited reproducibility of the content produced by both AI and human approaches, with the former being dependent on the prompts and software version used as well as changing online content, which can be limited but not eliminated by ring-fencing sources, whilst the latter is also time and context-dependent but also influenced by external factors to a greater extent, such as the quality of facilitation.^25^, ^26^ These limitations are inherent to a postgraduate training environment as well as broader clinical practice and thus need to be factored into any educational intervention design. Lay representatives were engaged as an alternative to patient input due to the material produced being explicitly aimed at doctors’ skills development, however, any educational interventions that were more specialty- or locality-oriented would need to be contextualised by taking into account local population needs, clinical practice, participant knowledge, skill levels and patient and learner feedback.

In determining whether AI might positively augment educational interventions in sensitive areas such as BBN, we advocate that it can, provided that AI-generated content is carefully curated and integrated with existing best practice in clinical supervision and reflection. Used in this manner, it may enhance the development of skills in breaking bad news from generic to more specialist levels.

## Conclusions

This study compared the teaching content generated by AI to human-facilitated discussion from a mixed methods perspective, using quantitative and qualitative data to highlight commonalities and exploring the merits of their differences. It also compared the responses of Group 1 and 2 physician specialties. Analysis revealed that structuring the content to the experience of the learner was critical and that elements of both human and AI approaches might be used to optimal effect to all physician specialties, for example, depending on whether a more basic (e.g. stepwise approach with specific examples) or sophisticated approach (e.g. encouraging the learner to develop their own personal style) was appropriate. Both approaches complemented the use of existing popular BBN protocols, such as SPIKES, with an additional focus on supporting doctors’ reflection post-event. Applied in this context, by adding to the range of learning styles and tailored content available, AI may help advance the development of more specialist skills in BBN, especially when supported by regular clinical supervision and reflective practice.

## CRediT authorship contribution statement

**David Marshall:** Writing – review & editing, Validation. **Michael C. Jones:** Writing – original draft, Validation, Resources, Methodology, Conceptualization. **Miriam E. Armstrong:** Writing – original draft, Resources, Project administration, Methodology, Investigation, Formal analysis, Data curation, Conceptualization. **Philip Bright:** Writing – review & editing, Validation, Methodology, Conceptualization. **Stephen Glen:** Writing – review & editing, Validation.

## Ethics approval and consent to participate

The Federation of the Royal Colleges of Physicians of the United Kingdom has a Research Ethics Committee which considers potential research applications involving data produced by its normal business processes. Its policies do not require formal ethical approval for Quality Improvement/Audit projects or surveys where participation is voluntary, consent is explicitly obtained and comments are non-attributable. No formal ethics approval was required as all participation in the online consultation was voluntary with full disclosure of the aims of the project, including the evaluation and use of data, discussed with Specialty Advisory Committee Chairs and Heads of Schools of Postgraduate Medicine before the project began and at the beginning of subsequent internal committee meetings. Participants taking part in the online survey were reminded of the purposes of the collection and use of the data, including that comments would be non-identifiable, at the beginning of the survey. They were explicitly asked to confirm they consented to their data being used for research purposes at the end of the survey (see supporting material). No attributable comments or data have been included in the article.

## Funding

This research did not receive any specific grant from funding agencies in the public, commercial, or not-for-profit sectors.

## Declaration of competing interest

The authors declare that they have no known competing financial interests or personal relationships that could have appeared to influence the work reported in this paper.

## Data Availability

The authors confirm that the data supporting the findings of this study are available within the article and its supplementary materials.
