# Supplementary material for: Evaluating the use of artificial intelligence and human guidance to teach generic and specialist skills in breaking bad news: A mixed methods exploration
Source: Clin Med (Lond). 2026 Sep 28;26(6):100649. doi: 10.1016/j.clinme.2026.100649 (PMC13677254; doi:10.1016/j.clinme.2026.100649)
Supplement: Supplementary file 1 [file mmc3.pdf]

What is the best way to break bad news?
Testing different approaches

Introduction

As part of its wider Equality, Diversity and Inclusion (EDI) work, the Joint Royal Colleges of Physicians Training Board (JRCPTB) is trialling new approaches to developing Generic Professional Capabilities (GPCs) or Capabilities in Practice (CiPs) for resident doctors.

You are invited to comment on guidance for 'breaking bad news' produced using non-traditional medical training approaches.

We are seeking feedback on its value for patients (including lay representatives) and doctors from different specialties. The findings will be reviewed together at an online workshop in early 2025. Please enter a contact email address at the end of the survey if you would like to take part in the workshop.

The questionnaire should take around 15 minutes to complete. There are three sections:

1. About you

2. Version A of the guidance

3. Version B of the guidance.

The questionnaire is anonymous (unless you choose to enter a personal email address at the end). All results will be presented collectively (with no individual identification) therefore please answer as fully as you can. More information on how the JRCPTB manages data is available here.

Any questions can be directed to Miriam.Armstrong@thefederation.uk.

Thank you for taking part.

Section 1 - About you

This information helps us understand how people experience clinical situations and / or training guidance differently.

* 1. Which of the following best describes your main area of work?

* 2. Which of the following best describes your national identity?

* 3. If you have an undergraduate medical degree (primary medical qualification) was this obtained in the UK?

- ◯ Yes
- ◯ No
- ◯ Don't know or Not applicable

* 4. What is your age?

- ◯ 30 years or under
- ◯ 31 - 40 years (inclusive)
- ◯ 41 - 50 years (inclusive)
- ◯ 51 - 60 years (inclusive)
- ◯ 61 years or older
- ◯ Prefer not to say

* 5. Which of the following best describes your ethnicity?

- ◯ White
- ◯ Mixed
- ◯ Asian
- ◯ Black
- ◯ Other ethnic group
- ◯ Prefer not to say

* 6. Are you:

- Male?
- Female?
- Identify in some other way?
- Prefer not to say

* 7. Do you have a disability, long-term illness or health condition?

- Yes
- No
- Prefer not to say

Section 2 - Version A

The following series of statements were produced as guidance for a medical training environment in response to the question: What is the best way to break bad news?

Participants are asked to rate how much they agree, or disagree, with each statement – with the option to provide further comments as required.

* 8. *Preparation:*

It is critical to not make the experience more upsetting than it needs to be, for both doctor and patient. Even five minutes spent familiarising yourself with the patient, so you can establish a relationship of sorts, is better than nothing.

Strongly agree	Agree	Neither agree nor disagree	Disagree	Strongly disagree	Prefer not to say or N/A
◯	◯	◯	◯	◯	◯

Comments (optional):

* 9. *Age of the patient:*

The age of the patient really matters to the approach. Final relief of suffering for elderly patients is very different to an unexpected or young death or where it has a significant household impact.

Strongly agree	Agree	Neither agree nor disagree	Disagree	Strongly disagree	Prefer not to say or N/A
◯	◯	◯	◯	◯	◯

Comments (optional):

* 10. *Find the right location:*

Private space is important whether a side-room, or corner of the bed, depending on the mobility of the patient or family. Purposively moving the discussion to a private area sends non-verbal signals that there is significant news.

Strongly agree	Agree	Neither agree nor disagree	Disagree	Strongly disagree	Prefer not to say or N/A
◯	◯	◯	◯	◯	◯

Comments (optional):

* 11. *Demonstrate empathy:*
Being able to demonstrate empathy through words and body language should be part of preparation, as well as being emotionally prepared for the task.

Strongly agree	Agree	Neither agree nor disagree	Disagree	Strongly disagree	Prefer not to say or N/A
○	○	○	○	○	○

Comments (optional):

* 12. *Previous history:*
What were the expectations previously given to the patient – were they realistic? Giving false hope does not help.

Strongly agree	Agree	Neither agree nor disagree	Disagree	Strongly disagree	Prefer not to say or N/A
○	○	○	○	○	○

Comments (optional):

* 13. *Tailor information to the patient:*
Breaking bad news ‘well’ can have a relieving, if not satisfying, effect. This is with the knowledge that you have made a positive contribution, even in a very difficult situation.

Strongly agree	Agree	Neither agree nor disagree	Disagree	Strongly disagree	Prefer not to say or N/A
○	○	○	○	○	○

Comments (optional):

* 14. *Humanity is key:*
Showing some kindness and humanity is fundamental.

Strongly agree	Agree	Neither agree nor disagree	Disagree	Strongly disagree	Prefer not to say or N/A
○	○	○	○	○	○

Comments (optional):

* 15. *Reflect on the response:*
The patient, or family’s, response can have a significant impact on the doctor - whether it was tearful, despairing, aggressive, in denial, completely unemotional or just ‘thank you’.

Strongly agree	Agree	Neither agree nor disagree	Disagree	Strongly disagree	Prefer not to say or N/A
○	○	○	○	○	○

Comments (optional):

* 16. *Reflect on any problems:*
Dealing with an abusive response can be debilitating for the doctor when effort has been taken to prepare for the situation as well as possible, but the response is still aggressive or critical.

Strongly agree	Agree	Neither agree nor disagree	Disagree	Strongly disagree	Prefer not to say or N/A
○	○	○	○	○	○

Comments (optional):

* 17. *Take time between patients:*
Managing the aftermath of breaking bad news can be difficult – especially when having to move onto the 'next patient' and giving them positive news in comparison.

Strongly agree	Agree	Neither agree nor disagree	Disagree	Strongly disagree	Prefer not to say or N/A
○	○	○	○	○	○

Comments (optional):

* 18. *Practise self-awareness:*
Being self-aware and prepared every time you have to break bad news is important to long-term wellbeing.

Strongly agree	Agree	Neither agree nor disagree	Disagree	Strongly disagree	Prefer not to say or N/A
○	○	○	○	○	○

Comments (optional):

* 19. *Seek additional support:*
Both peer and senior support is helpful both before and after the event, to be able to debrief and move on to the next patient having done the best job possible for each.

Strongly agree	Agree	Neither agree nor disagree	Disagree	Strongly disagree	Prefer not to say or N/A
○	○	○	○	○	○

Comments (optional):

* 20. *Choose 'reflection colleagues' carefully:*
The personality of peers and senior colleagues will determine whether you can ask them for help on non-clinical issues. Be aware that issues might resurface longer-term and know who to ask for help in managing them.

Strongly agree	Agree	Neither agree nor disagree	Disagree	Strongly disagree	Prefer not to say or N/A
○	○	○	○	○	○

Comments (optional):

* 21. *Team members:*
If senior doctors are asking more junior staff to assist in breaking bad news, they need to check they are ok to do this in advance.

Strongly agree	Agree	Neither agree nor disagree	Disagree	Strongly disagree	Prefer not to say or N/A
○	○	○	○	○	○

Comments (optional):

22. Please enter any additional thoughts or comments on Version A here:

Section 3 - Version B

The following series of statements were produced as guidance for a medical training environment in response to the question: What is the best way to break bad news?

Participants are asked to rate how much they agree, or disagree, with each statement – with the option to provide further comments as required.

* 23. *Understand the situation:*
Ensure you fully understand the patient's condition, prognosis, and the details of the bad news. This preparation allows you to answer any questions the patient or family may have.

Strongly agree	Agree	Neither agree nor disagree	Disagree	Strongly disagree	Prefer not to say or N/A
○	○	○	○	○	○

Comments (optional):

* 24. *Mentally prepare:*
Take a moment to compose yourself, recognising the emotional weight of the task. This helps you stay calm and present during the conversation.

Strongly agree	Agree	Neither agree nor disagree	Disagree	Strongly disagree	Prefer not to say or N/A
○	○	○	○	○	○

Comments (optional):

* 25. *Private and quiet environment:*
Choose a private, quiet room where you won't be interrupted. This ensures the patient or family feels respected and can process the information without distractions.

Strongly agree	Agree	Neither agree nor disagree	Disagree	Strongly disagree	Prefer not to say or N/A
○	○	○	○	○	○

Comments (optional):

* 26. *Involve support:*
If possible, have a nurse, social worker, or a more experienced physician present for support. This can help manage the conversation and provide additional comfort to the patient.

Strongly agree	Agree	Neither agree nor disagree	Disagree	Strongly disagree	Prefer not to say or N/A
◯	◯	◯	◯	◯	◯

Comments (optional):

* 27. *Start with open-ended questions:*
Ask the patient what they understand about their condition. This helps you gauge their knowledge and expectations, allowing you to tailor your communication.

Strongly agree	Agree	Neither agree nor disagree	Disagree	Strongly disagree	Prefer not to say or N/A
◯	◯	◯	◯	◯	◯

Comments (optional):

* 28. *Determine how much they want to know:*
Some patients prefer detailed information, while others might want only the essentials. Respect their preferences.

Strongly agree	Agree	Neither agree nor disagree	Disagree	Strongly disagree	Prefer not to say or N/A
◯	◯	◯	◯	◯	◯

Comments (optional):

* 29. *Use simple language:*
Avoid medical jargon. Use clear, straightforward language to explain the situation. This helps ensure that the patient or family fully understands the news.

Strongly agree	Agree	Neither agree nor disagree	Disagree	Strongly disagree	Prefer not to say or N/A
◯	◯	◯	◯	◯	◯

Comments (optional):

* 30. *Be direct but gentle:*
Clearly state the bad news, but do so in a compassionate tone. For example, instead of saying, "The cancer has metastasised," you might say, "I'm afraid I have some difficult news. The cancer has spread to other parts of your body."

Strongly agree	Agree	Neither agree nor disagree	Disagree	Strongly disagree	Prefer not to say or N/A
◯	◯	◯	◯	◯	◯

Comments (optional):

* 31. *Pause and listen:*
After delivering the news, pause to give the patient or family time to process. Listen actively to their responses, concerns, and emotions.

Strongly agree	Agree	Neither agree nor disagree	Disagree	Strongly disagree	Prefer not to say or N/A
◯	◯	◯	◯	◯	◯

Comments (optional):

* 32. *Recognise emotions:*
Acknowledge and validate the patient’s or family’s emotions. Phrases like "I can see this is very upsetting" or "I’m sorry to have to share this news" show empathy and understanding.

Strongly agree	Agree	Neither agree nor disagree	Disagree	Strongly disagree	Prefer not to say or N/A
◯	◯	◯	◯	◯	◯

Comments (optional):

* 33. *Provide emotional support:*
Offer reassurance that you will continue to support them through the next steps. This helps build trust and provides comfort.

Strongly agree	Agree	Neither agree nor disagree	Disagree	Strongly disagree	Prefer not to say or N/A
◯	◯	◯	◯	◯	◯

Comments (optional):

* 34. *Offer a plan:*
After the news is delivered, discuss the next steps in care or treatment. This might include further tests, treatment options, or palliative care. Providing a plan helps the patient or family focus on what can be done moving forward.

Strongly agree	Agree	Neither agree nor disagree	Disagree	Strongly disagree	Prefer not to say or N/A
◯	◯	◯	◯	◯	◯

Comments (optional):

* 35. *Answer questions:*
Be prepared to answer questions and clarify any doubts. It's important to be honest, but also sensitive to the patient’s emotional state.

Strongly agree	Agree	Neither agree nor disagree	Disagree	Strongly disagree	Prefer not to say or N/A
◯	◯	◯	◯	◯	◯

Comments (optional):

* 36. *Offer written materials:*
Provide written information that the patient or family can review later. This is particularly helpful as they may not remember everything discussed during the conversation.

Strongly agree	Agree	Neither agree nor disagree	Disagree	Strongly disagree	Prefer not to say or N/A
◯	◯	◯	◯	◯	◯

Comments (optional):

* 37. *Refer to support services:*
If available, refer the patient or family to counselling services, support groups, or social workers who can provide additional support.

Strongly agree	Agree	Neither agree nor disagree	Disagree	Strongly disagree	Prefer not to say or N/A
◯	◯	◯	◯	◯	◯

Comments (optional):

* 38. *Reflect on the experience:*
After the conversation, take time to reflect on how it went. Consider what went well and what could be improved for next time.

Strongly agree	Agree	Neither agree nor disagree	Disagree	Strongly disagree	Prefer not to say or N/A
○	○	○	○	○	○

Comments (optional):

* 39. *Seek feedback:*
If possible, seek feedback from a more experienced colleague who may have been present during the conversation. This can be invaluable in improving your skills in breaking bad news.

Strongly agree	Agree	Neither agree nor disagree	Disagree	Strongly disagree	Prefer not to say or N/A
○	○	○	○	○	○

Comments (optional):

* 40. *Acknowledge your own emotions:*
Delivering bad news can be emotionally taxing. It's important to acknowledge your own feelings and seek support if needed.

Strongly agree	Agree	Neither agree nor disagree	Disagree	Strongly disagree	Prefer not to say or N/A
○	○	○	○	○	○

Comments (optional):

* 41. *Debrief with colleagues:*
Discussing the experience with colleagues can help process the emotional impact and gain insights for future situations.

Strongly agree	Agree	Neither agree nor disagree	Disagree	Strongly disagree	Prefer not to say or N/A
○	○	○	○	○	○

Comments (optional):

42. Please enter any additional thoughts or comments on Version B here:

* 43. Overall, do you prefer Version A or Version B of the guidance?

Version A	Version B	Neither / neutral	Don't know / Prefer not to say
◯	◯	◯	◯

* 44. I consent for the information collected to be used for research and development or quality improvement purposes.

◯ Yes

◯ No

45. Please enter an email address if you would like to be invited to take part in an online workshop to discuss the findings:

46. If you would like to take part in an online workshop, which date would you prefer?
A date that suits the most respondents will be confirmed after the survey closes at 12.00 Monday 20 January

14.00 - 15.30 Monday 17 February	14.00 - 15.30 Monday 3 March	Both dates are fine	Neither date is suitable
◯	◯	◯	◯

Are there any other comments?

THIS IS THE END OF THE QUESTIONNAIRE. THANK YOU FOR TAKING PART.

IF YOU HAVE ANY QUESTIONS PLEASE CONTACT: Miriam.Armstrong@thefederation.uk.
