# Supplementary material for: Evaluating the use of artificial intelligence and human guidance to teach generic and specialist skills in breaking bad news: A mixed methods exploration
Source: Clin Med (Lond). 2026 Sep 28;26(6):100649. doi: 10.1016/j.clinme.2026.100649 (PMC13677254; doi:10.1016/j.clinme.2026.100649)
Supplement: Supplementary file 2 [file mmc1.docx]

**SUPPLEMENTARY MATERIAL**

**Table showing respondents mapped to Group 1 and Group 2 physician specialty participants**

**Online consultation: What is the best way to break bad news?**

| **Specialty** | **Specialty type** | **Total number of participants**  **(quantitative assessment)** | **Free-text comments provided (qualitative assessment)** |
| --- | --- | --- | --- |
| Acute Internal Medicine | Group 1 | 3 | 2 |
| Allergy and Immunology | Group 2 | 1 | 0 |
| Audiovestibular Medicine | Group 2 | 2 | 2 |
| Cardiology | Group 1 | 3 | 2 |
| Clinical Genetics | Group 2 | 1 | 1 |
| Clinical Pharmacology | Group 1 | 1 | 1 |
| Endocrinology and Diabetes Mellitus | Group 1 | 2 | 2 |
| Gastroenterology | Group 1 | 1 | 0 |
| Genitourinary Medicine | Group 1 | 3 | 2 |
| Geriatric Medicine | Group 1 | 4 | 4 |
| Haematology | Group 2 | 2 | 2 |
| Infectious Diseases and Tropical Medicine | Group 1 | 2 | 1 |
| Internal Medicine | Group 1 | 2 | 1 |
| Medical Oncology | Group 2 | 22 | 8 |
| Paediatric Cardiology | Group 2 | 1 | 1 |
| Palliative Medicine | Group 1 | 8 | 7 |
| Pharmaceutical Medicine | Group 2 | 1 | 1 |
| Respiratory Medicine | Group 1 | 7 | 3 |
| Rheumatology | Group 1 | 2 | 1 |
| Lay representatives | N/A | 12 | 8 |
| **Total responding** |  | **Group 1 = 38**  **Group 2 = 30**  **80 participants including 12 lay reps**  **19 participating specialties** | **Group 1 = 26 (68%)**  **Group 2 = 15 (50%)**  **49 (61%) participants including 8 lay reps**  **17 participating specialties** |

**Group 1 and Group 2 physician specialties**

The Joint Royal Colleges of Physicians Training Board (JRCPTB) sets the curriculum and assessment standards for thirty physician specialties, which are divided into Groups 1 and 2 for training purposes.

Group 1 specialties contribute most directly to acute and general medical care. Doctors normally complete three years of Internal Medicine (Stage 1) training before entering their chosen specialty and continue with Internal Medicine (Stage 2) training (also known as General Internal Medicine, GIM) alongside their specialty, before completing their ‘dual training’ with Certificates of Completion of Training (CCTs) in both GIM and their chosen specialty.

Group 2 specialties do not require ongoing training in GIM. Doctors usually complete two years of Internal Medicine (Stage 1) training before entering their chosen specialty. They do not hold ‘dual accreditation’ as they are not awarded a CCT in GIM alongside their specialty CCT.

| **GROUP 1 SPECIALTIES** | **GROUP 2 SPECIALTIES** |
| --- | --- |
| Acute Internal Medicine  Cardiology  Clinical Pharmacology & Therapeutics  Endocrinology & Diabetes Mellitus  Gastroenterology  Genitourinary Medicine (GUM)  Geriatric Medicine  Infectious Diseases *  Neurology  Palliative Medicine  Renal Medicine  Respiratory Medicine  Rheumatology  Tropical Medicine *  General Internal Medicine **  Stroke Medicine / General Internal Medicine *** | Allergy  Audiovestibular Medicine  Aviation & Space Medicine  Clinical Genetics  Clinical Neurophysiology  Dermatology  Haematology  Immunology  Infectious Diseases (when dual with Medical Microbiology or Virology) *  Medical Oncology  Medical Ophthalmology  Nuclear Medicine  Paediatric Cardiology  Pharmaceutical Medicine  Rehabilitation Medicine  Sport and Exercise Medicine  Tropical Medicine (when dual with Medical Microbiology or Virology) *    Chemical Pathology ****  Clinical Oncology  Medical Microbiology  Medical Virology  Occupational Medicine  Intensive Care Medicine ***** |

* Infectious Diseases and Tropical Medicine are Group 1 specialties, except when dual training with Medical Microbiology or Virology, in which case they are Group 2.

** can be taken a single specialty but usually undertaken alongside another Group 1 specialty.

*** sub-specialty pathway.

**** Chemical Pathology is generally regarded as a pathology specialty rather than a physician specialty and is overseen by the Royal College of Pathologists. Training leads to a CCT in Chemical Pathology. Under the current curriculum, Metabolic Medicine is incorporated into Chemical Pathology training rather than being a separate dual-accreditation programme.

***** separate curriculum but accessible after Internal Medicine Training.
