# Supplementary material for: Evaluating the use of artificial intelligence and human guidance to teach generic and specialist skills in breaking bad news: A mixed methods exploration
Source: Clin Med (Lond). 2026 Sep 28;26(6):100649. doi: 10.1016/j.clinme.2026.100649 (PMC13677254; doi:10.1016/j.clinme.2026.100649)
Supplement: Supplementary file 3 [file mmc2.docx]

**SUPPLEMENTARY MATERIAL**

**Tables highlighting mixed methods approach to evaluation of free-text responses for ‘breaking bad news’ (BBN):**

A) Primary qualitative themes and sub-themes with rationale
B) Responses for Group 1, Group 2 specialties and lay representatives mapped to qualitative sub-themes.
A. **Primary qualitative themes and sub-themes with rationale**

| **Primary theme and rationale** | **Sub-themes with rationale and examples** |
| --- | --- |
| Patient experience – matters viewed from patients’ or relatives’ perspectives | Improving patient experience [PE] - e.g. tailoring information, pausing to check understanding |
| Professionalism – focus on optimal approach or specific learning considerations to improve doctors’ capabilities. | Workplace-based practice improvements [WP] - e.g. preparation for consultation  Workplace-related soft skills development [WS] - e.g. building rapport or relationships  Dealing with uncertainty or difficulty [DU] - e.g. managing uncertainty or difficulty well |
| Access to support and further training – guidance available to doctors to enhance learning or wellbeing. | Near-event reflection / debrief / wellbeing [RD] – e.g. debriefing with colleagues  Longer-term skills development [SD] – e.g. building on experience.  Training or study methods [TM] - e.g. language awareness, allowing for ambiguity |

B. **Responses for Group 1, Group 2 specialties and lay representatives mapped to qualitative sub-themes**

| **BBN TOPIC** | **APPROACH (human vs AI)** | **GROUP 1 SPECIALTY** | **GROUP 2 SPECIALTY** | **LAY PERSPECTIVE** |
| --- | --- | --- | --- | --- |
| *1. Prior to the discussion:*  Understand basics, such as prognosis and who is involved in care, before talking to patient. | Human statement:  It is critical to not make the experience more upsetting than it needs to be, for both doctor and patient. Even five minutes spent familiarising yourself with the patient, so you can establish a relationship of sorts, is better than nothing. | I think essential to try and gain some kind of rapport (ie who's with them, anyone important they want with them, how they are feeling etc).  (Palliative Medicine)  [PE]  Perhaps say "even five minutes spent familiarising yourself with the patient's case, and considering how you will approach the discussion, can make a big difference. The wording "establishing a relationship or sorts, is better than nothing" is also a bit negative. I would try to emphasise that doing some groundwork/ preparation will help the resident doctors confidence and give them a better chance of establishing a trusted relationship with the patient to facilitate a difficult conversation.  (Palliative Medicine)  [PE, WP]  I think it is always easier if you know the patient and family. Often, on-call doctors are asked to make challenging and complex decisions. Difficult - for the patients/relatives and the doctor as the relationship is limited.  (Palliative Medicine)  [PE]  Preparation is key, may take longer than 5 mins to prepare. Need to be clear on information shared and discussed.  (Palliative Medicine consultant)  [WP]  Preparation is key to breaking bad news.  (Palliative Medicine)  [WP]  Preparation is key and more than 5 minutes is needed.  (Geriatric Medicine)  [WP]  This depends on the patient's expectations (if they are expecting bad news, sometimes they want either reassurance or confirmation much earlier than 5 minutes. I totally agree that establishing rapport is important, but how long is spent should be guided on an individual basis.  (Respiratory Medicine)  [PE, WP]  I feel that the comment of even 5 minutes to familiarise yourself in a way justifies potentially entering a situation not fully understanding or appreciating the context which can be detrimental.  (Endocrinology & Diabetes)  [PE, WP]  Not sure about the phrasing here. 'Is better than nothing' is quite negative. Something like 'can make a difference' or 'has value' might be better.  (Clinical Pharmacology and Therapeutics)  [TM]  Quite context specific. Ideally would have pre-existing relationship, but sometimes bad news is your first interaction and the patient is desperate for results.  (Infectious Diseases & Tropical Medicine)  [PE] | Agree but it is not adequate. Trust is vital and not having a good understanding of the patient's case demeans trust.  (Medical Oncology)  [PE]  Very important to try and establish a rapport.  (Medical Oncology)  [PE]  Read the notes! And if you don’t know the patient yourself, find someone who does and find out about them before going anywhere near them.  (Paediatric Cardiology)  [PE, WP] | It’s not clear from the question whether this refers to talking to the patient to get to know them before you deliver the news. If it is that, then I think it’s perhaps more.  important that the patient sees a doctor they’ve seen before.  [PE, WP]  Talking to the patient for 5 minutes might actually be quite stressful for the patient who may be waiting for news as opposed to having a getting to know you chat.  [PE]  Familiarity with patient helps; don't jump into things.  [WP]  Person centred care needs to apply and taking that time is an indication of personal care and interest in the individual.  [PE] |
|  | AI statement:  Ensure you fully understand the patient's condition, prognosis, and the details of the bad news. This preparation allows you to answer any questions the patient or family may have. | Uncertainty is often a part of these conversations and being honest and open about this is important.  (Geriatric Medicine)  [DU]  Not always possible to know all details and prognosis.  (Acute Internal Medicine)  [DU] | Whilst, obviously, preparation is important, in practice this is not really practicable. A doctor in ED may need to break news of a suspected cancer diagnosis, but will not be able to provide significant detail about treatment or prognosis. There is a risk of doing harm by providing inaccurate information. Patients react badly to being told different things from different doctors.  (Medical Oncology)  [PE, WP]  Ideal but not always possible especially if in situation where "caught out" in giving bad news that was not expected within the consultation.  (Medical Oncology)  [DU]  Very important.  (Medical Oncology)  [PE]  Sometimes you may not have all the information and the patient needs referring on to a specialist. Information you give might be more about what to expect from the referral process.  (Haematology)  [DU, WP]  Need to understand something about the patient themselves as well.  (Paediatric Cardiology)  [PE] | So important. I know of too many instances when patients have found the doctor doesn’t appear to have any knowledge of their history. Very frustrating.  [PE] |
| 2. *Age of the patient:*  Tailor approach to the age of the patient. | Human statement only:  The age of the patient really matters to the approach. Final relief of suffering for elderly patients is very different to an unexpected or young death or where it has a significant household impact. | Whilst age is important, I think it is more related to the scenario and individual characteristics of the patient and those closest to them.  (Palliative Medicine)  [PE]  I think this is true societally and maybe on a population perspective but undoubtedly on individual nature impact to the individual patient and their loved ones can be felt the same.  (Palliative Medicine)  [PE]  I think consideration of age of the patient is very important in breaking bad news, but it should always be individualised. Stereotypes based on demographics or personal information such as age coupled with people’s bias/ assumptions are really dangerous and I think this style of question feeds into those stereotyped perceptions. The second sentence in particular, where it suggests that elderly patients deaths do not have a significant household impact, feeds into the societal view of the elderly being forgotten and their lives seen as “not as worthy”.  (Palliative Medicine)  [PE]  You should never make assumptions about the impact of the news, whereas it is true that for many elderly patients the news may be less devastating than to someone with a young family or unexpected, every episode of breaking bad news needs to be individualised and sensitively handled.  (Palliative Medicine)  [PE]  Some elderly patients are as devastated by life changing diagnoses as young patients - it is a very individual thing. In young patients there may be more considerations, such as finances, young family etc. that need to be considered - but it is very individual and shouldn't change the approach.  (Palliative Medicine)  [PE]  This is person dependent, not age dependent.  (Palliative Medicine)  [PE]  We should not presume to anticipate a patient's response to bad news, just based on age. Again the approach should be personalised, based on initial response to the assessment of expectations and response to a warning shot.  (Respiratory Medicine)  [PE]  Let's not use the term elderly, whilst the death of an older person who has perhaps been unwell for a long time and their death has been expected it may be as painful and distressing to family as that of a younger person. Everybody deserves a sensitive and tailored approach.  (Geriatric Medicine)  [PE, TM]  I think it is very individual. More based on individual experience and symptom profile than age alone. Sometimes we get stuck on the idea of a good innings versus an early loss.  (Geriatric Medicine)  [PE]  This is ageist surely? Approach may be situational but not dictated by age.  (Rheumatology consultant)  [PE, TM]  I don't think it's always age, more the overall context. Young patients can also have prolonged suffering from debilitating conditions. Changing 'age' to 'full circumstances' and removing 'elderly' would reflect my personal opinion on this.  (Clinical Pharmacology & Therapeutics)  [TM]  My broad approach would be similar though specifics may vary.  (Infectious Diseases & Tropical Medicine)  [WP] | Although this is a very broad statement and doesn't mean that elderly patients are not upset to be told of a terminal diagnosis.  (Medical Oncology)  [PE]  Age is usually important, but this cannot be a one size fits all approach.  (Medical Oncology)  [PE, WP]  Although does depend on the patient.  (Paediatric Cardiology)  [PE]  Every case is individual and you cannot make a sweeping statement about age affecting the consultation.  (Haematology)  [PE]  I agree the age of the patient matters re the approach and language used. However, at the expense of generalising, the impact on relatives can be devastating at any age although older patients may be more accepting of a bad news diagnosis.  (Audiovestibular Medicine)  [PE] | Context and knowing the patient is most important. You can’t assume an elderly person or their family will be ‘relieved’ by the prospect of, or actual death.  [PE]  Age MAY be an important consideration.  [PE] |
| 3. *Find the right location:*  Private and quiet environment is essential. | Human statement:    Private space is important whether a side-room, or corner of the bed, depending on the mobility of the patient or family. Purposively moving the discussion to a private area sends non-verbal signals that there is significant news. | Privacy and confidentiality should be prioritised whether it is good or bad news.  (Palliative Medicine)  [PE, WP]  Agree privacy is important.  (Palliative Medicine)  [PE, WP]  I think privacy is important to allow more open discussion. The primary intent is not necessarily to send non-verbal signals of significant news and wouldn't give this as a reason.  (Respiratory Medicine)  [PE]  I am not sure the corner of a bed is appropriate to breaking bad news unless there are absolutely no other options. These important and sensitive discussions should take place in private space -the non verbal signals about significant news can be important.  (Geriatric Medicine)  [PE, WP] | Private rooms not always available however.  (Medical Oncology)  [PE, WP]  Agree but not always possible or practical.  (Medical Oncology)  [PE, WP]  Agree but increasingly unrealistic for inpatients due to space constraints.  (Medical Oncology)  [PE, WP]  This is very important but often very difficult to arrange as NHS estate often does not provide suitable space for breaking bad news.  (Medical Oncology)  [PE, WP]  Agree with first sentence, disagree with second.  (Medical Oncology)  [PE, WP]  Just remember that curtains have ears!  (Paediatric Cardiology)  [PE, WP]  But guidance should also cover what to do when there is no access to a good location - busy ward, unexpectedly meeting relative in corridor, things you can't plan for.  (Haematology)  [TM] | It’s important to provide patients and their families with the  privacy and dignity of a safe and confidential space to hear the news and react to it.  [PE, WP]  I absolutely agree that moving the discussion to a private area gives a big non-verbal signal. When my son was in ITU for some months, the private room next to the waiting room was actually quite a scary prospect because you know you’re going to get good or bad news.  [PE, WP] |
|  | AI statement:  Choose a private, quiet room where you won't be interrupted. This ensures the patient or family feels respected and can process the information without distractions. | Practicalities of this can be challenging given the pressurised clinical environment.  (Cardiology)  [WP]  Not always possible in current state of NHS and acknowledgement of this is important.  (Acute Internal Medicine)  [WP]  Though personal safety is also key.  (Genitourinary Medicine)  [WP, TM] | Whilst this is preferable, it is not always practically achievable. Patients may be bedbound, or no private room available.  (Medical Oncology)  [PE, WP]  Where this is possible. Mobility may be an issue for the patient and the best option available should be used rather than finding the perfect room for their family and excluding them.  (Medical Oncology)  [PE, WP]  Sometimes this can be challenging in a ward environment.  (Medical Oncology)  [WP]  Ideal, not always possible.  (Medical Oncology)  [WP]  Needs to be recognised that this is not always possible.  (Medical Oncology)  [WP]  But don’t put it off if ideal location cannot be found,  (Medical Oncology)  [PE, WP]  But may not always be possible given the exigencies of the NHS.  (Paediatric Cardiology)  [WP]  Include what to do if nowhere quiet.  (Haematology)  [TM] |  |
| 4. *Emotional preparation:*  Accept seriousness of situation and need to maintain composure throughout. | Human statement:  Being able to demonstrate empathy through words and body language should be part of preparation, as well as being emotionally prepared for the task. | Yes absolutely. I think sometimes training in communication should go beyond how you say it, but also recognising reactions and developing understanding of those delivering bad news about how you say it and how not to respond to a distressed reaction.  (Palliative Medicine)  [TM, WS]  Empathy is critical but the manner in which this is expressed will need to be balanced and specific to circumstances. One size does not fit all.  (Respiratory Medicine)  [WS, PE]  Don't really understand what 'part of preparation' means here. As in providing a warning shot to patient / family, part of training, ensuring you are feeling up to that individual task.  (Clinical Pharmacology & Therapeutics)  [TM, WS] | Demonstrating empathy will differ for all of us, being more natural in some cases or depending on the link between those having the conversation. So while very important we have to accept it may not always be possible to achieve.  (Medical Oncology)  [WS]  Empathy isn’t just body language or words, it’s about listening, hearing and making the patient feel included and that their views matter.  (Paediatric Cardiology)  [WS]  Being aware of own limitations - tired and stressed doctors may find this more difficult, situations too close to home can be unexpectedly upsetting.  (Haematology)  [WS, RD]  Demonstration of empathy is an area which I find is challenging to some of our trainees who struggle to communicate outside of medical jargon.  (Audiovestibular Medicine)  [WS] | Doctors will need support to understand what this looks like in practice, and some may need more support than others. e.g. some types of neurodiversity may impact empathy.  [WP, WS, SD]  For more [MOST?] patients still hard. [NOT CODED]  'Demonstrate' is problematic; 'be empathic' is important; how to BE emotionally prepared? Ways of preparing ...  [TM, WS] |
|  | AI statement:  Take a moment to compose yourself, recognising the emotional weight of the task. This helps you stay calm and present during the conversation. | Thinking through potential queries / questions/ challenges is important.  (Geriatric Medicine)  [WP] | Yes and seek help from colleagues, consider asking a nurse specialist etc to be present.  (Medical Oncology)  [WP, WS]  Never rush it, but equally don't keep putting it off.  (Haematology)  [WP]  Seems reasonable but there is a bit more to it than that.  (Paediatric Cardiology)  [WP, WS] |  |
| 5. *Involve support:*  Additional support from co-workers. | AI statement only:  If possible, have a nurse, social worker, or a more experienced physician present for support. This can help manage the conversation and provide additional comfort to the patient. | Disagree about having more experienced physician there - this can be confusing for relatives / family. Nurse support absolutely. Social worker - never seen this in clinical practice over 30 years.  (Cardiology)  [TM, WP]  This will depend on the circumstances and any pre-existing relationship of the doctor with the patient and their family. Nursing and social support always welcome but not necessarily a senior colleague if the doctor is comfortable with the activity.  (Respiratory Medicine)  [TM, WP, PE]  Ideally needs to be somebody who will have more direct contact with the patient when doctor leaves - usually why nurses are better option than a more senior.  (Rheumatology)  [TM, WP, PE] | Why define what professions - suggest another colleague, it could be a physiotherapist etc who knows patient/ family.  (Medical Oncology)  [TM, WP, PE]  Yes I think wherever possible.  (Medical Oncology)  [WP]  Not always possible or realistic.  (Medical Oncology)  [TM, WP]  This depends on the patient. Ask them if they want anyone with them when you discuss their condition. Personally I'd be really cross if you wheeled someone in to "hold my hand" and make sympathetic noises with their head on one side and several of my patients have told me they hated this also.  (Haematology)  [TM, WP, PE]  ideally also have the patient have a family member or friend present to help them remember what was said.  (Paediatric Cardiology)  [WP, PE] | I know from experience how helpful this is both to the patient and no doubt the doctor.  [WP, PE]  It is not the task to provide comfort.  [TM, WP, PE]  Consider if it might be helpful to have ....  [TM]  As a patient I would find it more difficult to process the news with more than one person in the room (i.e. a watcher?)  [TM, WP, PE] |
| 6. *Assess engagement:*  Tailor communication to their knowledge and understanding. | Human statement:  What were the expectations previously given to the patient – were they realistic? Giving false hope does not help. | Sometimes breaking bad news is a process and not a single discussion allowing patients and families to have information of a depth and quality at a rate that suits them. Clarifying understanding and expectations at the start of any conversation is vital.  (Palliative Medicine)  [WP, PE]  A lot of emphasis is put in not giving false hope, but I think patients and their families will often want to keep hope until very late on as part of a coping strategy. I can understand why responsible teams don't want to always be blunt when faced with this, and sometimes not taking away hope is important. Offering to be honest maybe is the better approach and seeing what the response from that question is.  (Palliative Medicine)  [TM, WP, PE]  This helps frame the conversation in a supportive way, also can highlight if there is previous mis-communication / mis-perception and helps with continuity of communication and information.  (Palliative Medicine)  [WP]  This should be included in the early parts of the discussions with the patient and family.  (Palliative Medicine)  [WP, PE]  Often, cancer patients receive lots of news regarding an active treatment plan but not much about what happens should they deteriorate.  (Geriatric Medicine)  [WP, PE]  This knowledge is important in anticipation of questions that may arise and is a source of subsequent complaints if not prepared.  (Respiratory Medicine)  [WP, PE]  Presume this means identifying patients’ current knowledge - this is helpful on many levels.  (Rheumatology)  [WP, PE] | Exploring patient's previous understanding and expectations before breaking bad news can be very helpful in managing the process, but all patients are different and come from different cultural backgrounds and belief systems, some prefer to hold onto 'false hope' until the very end and need to be given bad news in a sensitive way - not everyone wants to know all the details.  (Medical Oncology)  [WP, PE]  Personal and cultural factors here at least, agree false hope instinctively is a bad thing from my perspective but "hope" may be important at stages in a patients journey and being "false" could be qualitative.  (Medical Oncology)  [WP, PE]  Patients can take false hope from many statements.  (Medical Oncology)  [DU]  Although in practice this can be difficult to negotiate.  (Medical Oncology)  [WP]  Too often I hear cancer cured.  (Pharmaceutical Medicine)  [WP, PE] | You need to be sure there is no hope - because hope is an important bit of kit that can help patients manage their wellbeing and quality of life.  [PE, WP]  OK to find the place the patient might be at, however, important not to give false hope in any circumstances.  [PE, WP]  Can be hard to predict how things will turn out; patients hear what they want to hear.  [DU]  Building on previous conversations.  [WP]  First part not important anymore.  [PE] |
|  | AI statement:  Ask the patient what they understand about their condition. This helps you gauge their knowledge and expectations, allowing you to tailor your communication. | Though sometimes people know you are going to deliver it and want you to get to the point.  (Palliative Medicine)  [WP, PE]  Depending how well you know the patient- sometimes this can come across as patronising for some patients depending how it is phrased as well.  (Palliative Medicine)  [WP, WS, PE]  Depends on situation - may not be appropriate - presume this is about telling patient bad news rather than telling family of patient's death for example.  (Cardiology)  [WP]  I think this depends on the circumstances. In my experience this can be viewed as 'beating around the bush' by some patients.  (Respiratory Medicine)  [WP, PE]  This is by far the most important part of the interaction in my view.  (Infectious Diseases & Tropical Medicine)  [WP, PE] | Not always necessary - and can irritate some patients when they've already answered questions with other staff, although a degree of this is always important.  (Medical Oncology)  [WP, PE]  Find out what they know first and what they are worried about then start building on that and filling gaps - but don't let them derail the conversation.  (Haematology)  [WP, PE] | Caveat is that this may come across as a bit of an interrogation!  [WP, PE]  It may sometimes be helpful to ...  [TM]  For all patients.  [TM, PE] |
| 7. *Respect patient preferences:*  Be guided by the patient’s preferences to receive information. | Human statement:  Breaking bad news ‘well’ can have a relieving if not satisfying effect. This is with the knowledge that you have made a positive contribution, even in a very difficult situation. | The most important thing is to pace information for an individual patient - and keep checking what they want to know. Some patients never want to know bad news and it can be harmful to force it - so pacing and tailoring is critical. We also teach doctors to summarise as they go and at the end, checking understanding as they go. Then given an opportunity for a follow up for more questions.  (Palliative Medicine)  [WP, PE]  Focus should remain on the patient and their family.  (Palliative Medicine)  [TM, PE]  Agree in part. I do not see this activity as a positive contribution. It is more important a case of not doing it badly.  (Respiratory Medicine)  [TM]  Even the worst jobs can be done well. Knowing it was done as well as possible limits the moral injury to the doctor.  (Rheumatology)  [WP, RD] | Breaking bad news well in varying situations is a difficult skill and takes a lot of experience and practice, it is a rewarding experience when it goes well.  (Medical Oncology)  [WP, RD]  "Tailoring information" is not as important as giving necessary information well.  (Medical Oncology)  [WP, WS]  I am surprised that this 'about me' is so early a statement.  (Medical Oncology)  [TM]  There can be significant negative impact on the mental wellbeing of the person conveying the news.  (Clinical Genetics)  [RD]  However upsetting it can be to deliver bad news it is better that it is you doing it well with compassion than someone else in a hurry doing it badly.  (Haematology)  [WP, PE] | Very important. This relies on knowing the patient.  [WP, PE]  'A relieving if not satisfying effect' - for whom? patient or doctor?  [TM, PE] |
|  | AI statement:  Some patients prefer detailed information, while others might want only the essentials. Respect their preferences. | Some people never want to know detail and may prefer news given to a friend or family member - this is OK!  (Palliative Medicine)  [WP, PE]  This is often a process -not just one conversation.  (Geriatric Medicine)  [WP, PE]  Sometimes you do need to be fairly precise (perhaps not blunt) - euphemisms can be "too gentle" (if there is such a way of putting it) and perpetuate misunderstanding of the gravity of the situation.  (Geriatric Medicine)  [WP, PE]  This is risky as interpretations can be wrong. If done properly patients will grasp the direction of conversation / guess and will ask or demonstrate how much info they want.  (Rheumatology)  [WP, PE] | Add that family may have different expectations and patient is the primary here.  (Medical Oncology)  [TM, PE]  I'm not sure patients always know what they want - a sensitive conversation taking cues from the patient and carefully exploring things is more important than this.  (Medical Oncology)  [WP, WS, PE]  You should be able to determine this by listening as you go through things; communication is 2 way!  (Paediatric Cardiology)  [WP, WS]  Always tricky to ask how much patients want to know, this is often something that is gauged by previous interactions with patient.  (Audiovestibular Medicine)  [WP, PE] | Can be difficult to determine this though. Some pointers on how to establish this might help.  [TM, WP]  Sometimes you break bad news and patient does not understand it.  [WP, PE]  Offer both by trying.  [WP, PE] |
| 8. *Use simple language:*  Avoid medical jargon. | AI statement only:  Avoid medical jargon. Use clear, straightforward language to explain the situation. This helps ensure that the patient or family fully understands the news. | Avoid euphemisms as well as jargon.  (Palliative Medicine)  [WP]  I think it is useful to explain in both medical and lay terms.  (Infectious Diseases & Tropical Medicine)  [WP] | For all conversations, not just breaking bad news.  (Medical Oncology)  [TM. WP]  Agree, and if death is involved then definitely use the D word not euphemisms.  (Paediatric Cardiology)  [WP] | Not all patients know the word 'malignant' means cancer.  [TM]  Use pictures, sketches.  [TM] |
| 9. *Pause and listen:*  Pause to assist communication. | AI statement only:  After delivering the news, pause to give the patient or family time to process. Listen actively to their responses, concerns, and emotions. | Pauses after each bit of news is very important. These may occasionally need to be prolonged to ensure the information has been understood.  (Respiratory Medicine)  [WS]  Active listening is a real skill.  (Geriatric Medicine)  [WS] | Telling people to pause if this is not something they can do is difficult. I sometimes say to "use silence", rather than pause, and not be afraid to not fill every second with noise. But artificial "pausing because I've been told to" is not necessarily useful. But absolutely listen to them.  (Medical Oncology)  [WS]  Not rushing through a prepared spiel is important, listening to reaction will help guide further approach.  (Medical Oncology)  [WS] | Really listen. Only answer to questions!  [WS] |
| 10. *Show compassion:*  Show some humanity. Clearly state the bad news but do so in a compassionate tone. | Human statement:  Showing some kindness and humanity is fundamental. | Whilst maintaining professionalism.  (Clinical Pharmacology & Therapeutics)  [WP] | And humility.  (Pharmaceutical Medicine)  [WS] | Doctors may need support to develop strategies for what this might look like in practice.  [TM, SD]  Is 'showing' enough?  [TM, SD] |
|  | AI statement:  Clearly state the bad news but do so in a compassionate tone. For example, instead of saying, "The cancer has metastasised," you might say, "I'm afraid I have some difficult news. The cancer has spread to other parts of your body." | However, remember if you have someone that really doesn't want to know and delegates information receiving to another, we need to respect this and then sense check regularly.  (Palliative Medicine)  [WP, PE]  Important to avoid jargon.  (Geriatric Medicine)  [WP] | This approach will depend on the patient and family their backgrounds and beliefs.  (Medical Oncology)  [WP, PE]  True but giving example is different to other stems and may not be required and is not related to compassionate tone rather a more compassionate sentence?  (Medical Oncology)  [TM]  Obviously.  (Medical Oncology)  [PE]  Also goes with 29. [NOT CODED]  (Paediatric Cardiology) | Excellent use of a practical example of what this looks like.  [TM] |
| 11. *Reflect on the response:*  Take some time to process emotional response. | Human statement only:  The patient, or family’s, response can have a significant impact on the doctor - whether it was tearful, despairing, aggressive, in denial, completely unemotional or just ‘thank you’. | An initial response can be misleading as the patient and or family may be shocked and need time to digest/ consider news. Many patients and families need to come back for more questions/ information. You need to be careful here that you are not suggesting to resident doctors that there is a "right" response.  (Palliative Medicine)  [TM]  Although the focus should remain on the patient, and doctors should be able to respond to the emotional response.  (Palliative Medicine)  [TM, WS, DU]  I think engagement with the family while maintaining personal detachment is important. The patient and family's response will always have some emotional impact which needs to be managed with perspective. Important that feedback is taken of the patient / family response to guide learning.  (Respiratory Medicine)  [WS, RD, SD]  I have had some awful interactions centred around you are giving-up etc. Need for support for the breaker of the bad news is important.  (Geriatric Medicine)  [RD, DU] | Doctor may receive a wide range of responses.  (Medical Oncology)  [DU]  This is a sign of maturity or lack of it as a doctor, the more experienced you become the less the patient impact is on you personally - it is our/your job and we are trained.  (Medical Oncology)  [DU]  Personal experience of family conflict – disturbing.  (Pharmaceutical Medicine)  [DU] | Emphasis is focusing on the patient - they should be given latitude and it might seem wrong if you are tempted to feel a victim following the situation.  [PE, DU, SD] |
| 12. *Provide emotional support:*  Offer reassurance. | AI statement only:  Offer reassurance that you will continue to support them through the next steps. This helps build trust and provides comfort. | May not be their role moving forwards.  (Acute Internal Medicine)  [TM, WP]  Not always possible or realistic.  (Cardiology)  [TM, WP]  Provided this information is accurate. If the patient's care will be handed over to someone else, then this should also be clear.  (Respiratory Medicine)  [WP]  May not be appropriate that individual offers ongoing support as they may not be involved in ongoing care.  (Geriatric Medicine)  [TM, WP]  If realistic. If not (patient moving ward / hospital etc) then giving a plan instead.  (Rheumatology)  [WP] | Careful as may not be able to deliver, will it be "you" that supports or other members of the team.  (Medical Oncology)  [TM, WP]  if relevant. If you're never going to see her patient or family again this is dishonest.  (Medical Oncology)  [TM, WP]  This is often the last contact you have with them as they will be passing to another team, so do not promise what you or your team cannot deliver.  (Medical Oncology)  [TM, WP]  And explain what the next steps are.  (Paediatric Cardiology)  [WP] | This can be difficult to promise with the fragmentation of care.  [TM, WP]  Only if you are really able to do this (not to relieve yourself or better the situation).  [TM, WP]  If it is true.  [TM, WP]  If you know you are in a position to do this.  [WP] |
| 13. *Allow for emotions*:  Acknowledge and validate the patient’s or family’s emotions. | Human statement:  Dealing with an abusive response can be debilitating for the doctor when effort has been taken to prepare for the situation as well as possible, but the response is still aggressive or critical | Reflecting why patients or families may be aggressive or critical is important and can be a response to stress / distress. However, it can also be when the communicating doctor is unprepared or doesn't leave a clear plan. I would add something in about the range of reactions patients and families may express and recognise the factors that might impact responses.  (Palliative Medicine)  [RD, WS, TM]  This is also a natural response to bad news and ideally should be handled by doctors in the same way as Q15.  (Respiratory Medicine)  [DU]  I think there has to be preparation for this unpredictable response and an awareness of why it may happen (anger at situation rather than individual). Can still be done well.  (Rheumatology)  [WP, WS]  Can be very difficult to move in with the shift. [NOT CODED]  (Infectious Diseases & Tropical Medicine) | it is hard when breaking bad news is done as empathically as possible and a complaint follows as the bad-news giver gets blamed for the bad news.  (Medical Oncology)  [DU]  Not debilitating - that is a bit strong for something that we do frequently.  (Medical Oncology)  [TM, DU]  Opportunity for debriefing when things don't go according to plan is crucial for learning, growth, resilience and emotional wellbeing of trainees.  (Medical Oncology)  [RD, DU]  Agree but understand that most anger comes from a place of fear in this situation.  (Haematology)  [DU]  Important trainees explore this and develop appropriate critical thinking as to the possible rationales, self reflection on breaking the news technique, and their subsequent resilience and coping strategies.  (Audiovestibular Medicine)  [RD, WS] | Difficult situation but not an uncommon reaction. Important medical professionals are taught to manage this both in the situation and afterwards to safeguard themselves.  [RD, SD]  Be aware of your own emotions.  [RD]  But to be expected.  [DU]  However it is important not to let this overwhelm the doctor.  [DU]  Response to 8 also applies here. [NOT CODED] |
|  | AI statement:  Acknowledge and validate the patient’s or family’s emotions. Phrases like "I can see this is very upsetting" or "I’m sorry to have to share this news" show empathy and understanding. | Depends on the patient! Have had mixed responses to the 'I can see...'  (Palliative Medicine)  [PE, WS]  Commentating on the emotions of the patient shouldn't always be volunteered. In my experience it is irritating for some patients and their families, particularly when they are surprised by the information.  (Respiratory Medicine)  [PE, WS]  If sensibly applied.  (Rheumatology)  [WS] | Adding specific examples might be taken out of context by less experience and then could be more inflammatory in a challenging consultation especially "I can see this is very upsetting".  (Medical Oncology)  [TM, WS]  But not everyone can do this and doing this artificially is worse than not doing it at all.  (Medical Oncology)  [TM, WS]  As long as it does not sound fake - and is natural for you - use your own words.  (Medical Oncology)  [WS]  And don't be surprised if you get "I'm sorry for you doctor having to tell me this" or "I couldn't do your job".  (Haematology)  [RD] | Good use of a practical example of what this might look like.  [TM]  it is not about the doctor.  [TM] |
| 14  *Offer a plan:*  More information regarding next steps. | AI statement only:  After the news is delivered, discuss the next steps in care or treatment. This might include further tests, treatment options, or palliative care. Providing a plan helps the patient or family focus on what can be done moving forward. | I think helpful to understand what you mean by palliative care. Do you mean end of life care? Do you mean symptom control?  (Palliative Medicine)  [TM]  It is likely that the patient may not remember all of these details so either offering written information about the plan, or a follow up visit / telephone call to discuss is also often useful.  (Respiratory Medicine)  [WP, PE]  Preparation for this part of the conversation should be done in advance with relevant social care and specialist nurses etc. Ideally they should also be present at the meeting.  (Respiratory Medicine)  [WP] | Wherever possible this is preferable, but it's also important that people delivering bad news restrict themselves to certainty, and don't offer plans that aren't confirmed.as this can lead to distress later. And I think it's important this be clarified in the guidance.  (Medical Oncology)  [WP, DU, TM]  Only if relevant / useful and the patient / family are in the right frame of mind to do this.  (Medical Oncology)  [WP, PE]  Often the end is 'you will see the next team who will explain all'...  (Medical Oncology)  [WP]  Bad news is not just you are dying and palliative care.... i don’t like the example it is not needed.  (Medical Oncology)  [TM]  But go at the patient's pace. Be wary of relatives who want to go faster than the patient.  (Haematology)  [WS] | There should be a break before a long talk about the plan.  [WP, PE]  At the right time, may require a pause.  [WP, WS]  Explain possible next steps.  [WP] |
| 15. *Answer questions:*  With honesty and tact. | AI statement only:  Be prepared to answer questions and clarify any doubts. It's important to be honest, but also sensitive to the patient’s emotional state. | If the doctor doesn't know the answer that is OK - need to be honest and explain who they will contact to help answer specific questions, not leave patients and families in limbo.  (Palliative Medicine)  [WP, WS, DU]  Preparation for this part of the conversation should be done in advance with relevant social care and specialist nurses etc. Ideally they should also be present at the meeting.  (Respiratory Medicine)  [WP]  Expressing uncertainty is ok as well.  (Rheumatology)  [DU] | Offer to return to discussion later as needed.  (Medical Oncology)  [WP, WS]  Unfortunately uncertainty is unavoidable at some points for us.  (Medical Oncology)  [DU] |  |
| 16. *Offer written materials:*  Provide written information for later review. | AI statement only:  Provide written information that the patient or family can review later. This is particularly helpful as they may not remember everything discussed during the conversation. | I have never done this. I don't know how helpful this is. Is there any evidence?  (Palliative Medicine)  [TM]  Written information that includes relevant contact numbers very important. If there is lot of information to process then may be better to arrange a further discussion soon, when the additional information is shared and discussed.  (Respiratory Medicine)  [WP]  The family may not want this and also need to be careful over exactly what is written and you have the tine and skills to do it well.  (Geriatric Medicine)  [WP, WS, PE]  Realistically not always available for patients’ individual situations - yes if available and directly relevant / validated.  (Rheumatology)  [WP]  Not always possible during a busy take shift.  (Internal Medicine doctor in training)  [WP] | May not be possible e.g. if conversation during ward round etc.  (Medical Oncology)  [WP]  If relevant - not everything has a leaflet.  (Medical Oncology)  [WP]  Not often done, but factual material is done.  (Medical Oncology)  [WP]  Yes where available.  (Medical Oncology)  [WP]  Where possible.  (Medical Oncology)  [WP]  This isn't always helpful and some people don't want the confirmation of the bad news in writing. However if this is the case, the information conveyed should be recorded contemporaneously in the patient's notes.  (Clinical Genetics)  [WP, PE]  Make sure you check the content of written information - patients will take it as gospel.  (Haematology)  [WP]  I would suggest providing a summary of what was discussed; either by hand or as a formal typed note at some stage that they can refer to.  (Paediatric Cardiology)  [WP] | Do it but don't force it.  [WP, PE] |
| 17. *Refer to support services:*  Counselling or other support services. | AI statement only:  If available, refer the patient or family to counselling services, support groups, or social workers who can provide additional support. | I think offering support services too quickly can also imply grief is an abnormal reaction when actually it is normal to feel upset etc. I think offering these when people are having pathological responses after allowing the info to settle in is better.  (Palliative Medicine)  [WP, WS]  Yes - but not necessarily immediately. It is normal for people to be upset and need time. They don't all need counselling!  (Palliative Medicine)  [WP]  Need to judge on individual basis.  (Geriatric Medicine)  [WP]  Where possible.  (Rheumatology)  [WP]  I have not worked in many places where this has been available, so in many settings will be redundant.  (Clinical Pharmacology & Therapeutics)  [WP] | If they consent.  (Medical Oncology)  [WP]  If relevant.  (Medical Oncology)  [WP]  Not always useful in that first conversation - can risk the patient feeling like you are just passing them on to someone else. Needs judgement of the right time to do it.  (Haematology)  [WP]  Needs to be done in a way that patients don't feel they are being fobbed off onto another service.  (Audiovestibular Medicine)  [WP, WS] | Sometimes this step should be repeated later.  [WP]  If the patient needs it.  [PE] |
| 18. *Take time to manage own response:*  Manage your own emotional response before moving to next patient. | Human statement:  Managing the aftermath of breaking bad news can be difficult – especially when having to move onto the ‘next patient’ and giving them positive news in comparison. | This will depend upon the how the doctor feels and the circumstances. Certainly shouldn't feel rushed to move on.  (Respiratory Medicine)  [RD] | This would ideally be possible, but rarely practical in busy clinical environments. Trainees should be given tools to reflect and debrief shortly after the event but be supported to continue with their clinical work in the immediate aftermath as other patients are relying on their care and ability to continue their duties.  (Medical Oncology)  [WP, RD]  That is the job.  (Medical Oncology)  [DU, WP]  Not easy in a resource restricted NHS.  (Medical Oncology)  [DU, WP]  But may not be realistic in a busy clinical setting, varies with individual giving the news.  (Medical Oncology)  [WP]  But in reality "taking time" between patients is not always possible.  (Medical Oncology)  [WP]  Learning to accept you are only human and it will affect you.  (Haematology)  [RD] |  |
|  | AI statement:  Delivering bad news can be emotionally taxing. It's important to acknowledge your own feelings and seek support if needed. |  | There will be certain triggers and times you are more vulnerable.  (Haematology)  [RD] | Vital.  [RD, DU] |
| 19  *Reflect on the experience:*  Consider what went well, what could be improved and own emotional impact.  . | Human statement:  Being aware of self and prepared every time you have to break bad news is important to long-term wellbeing. | Realising that you are ill prepared and things are going badly is a dreadful feeling.  (Infectious Diseases & Tropical Medicine)  [RD, DU] | Agree but not always possible - being aware of the pitfalls and the need to decompress are also important. Also self awareness to recognise your own personal trigger points.  (Haematology)  [RD, DU] | Your state impacts the discussion.  [RD, DU]  Isn't this just a fact?  [RD] |
|  | AI statement:  After the conversation, take time to reflect on how it went. Consider what went well and what could be improved for next time. |  | Yes reflect, but don't always aspire to making something better - sometimes it's enough to say you did ok.  (Medical Oncology)  [RD]  Good to discuss with team.  (Medical Oncology)  [RD] | If you have time.  [RD, WP] |
| 20. *Seek feedback:*  Seek feedback from peers or more experienced colleagues to improve skills. | Human statement:  Both peer and senior support is helpful both before and after the event, to be able to debrief and move on to the next patient having done the best job possible for each. | For doctors that have little or limited experience, this is very important.  (Respiratory Medicine)  [RD]  I have not had much experience of this happening, probably would be helpful but not commenting on this with any insight.  (Clinical Pharmacology & Therapeutics)  [NOT CODED] | Agree but again not easy within resource constraints.  (Medical Oncology)  [RD, WP] |  |
|  | AI statement:  If possible, seek feedback from a more experienced colleague who may have been present during the conversation. This can be invaluable in improving your skills in breaking bad news. | For more junior colleagues training in breaking bad news and getting feedback is part of this process.  (Geriatric Medicine)  [RD] | Yes, maybe, if relevant and needed.  (Medical Oncology)  [RD]  Feedback from a less experienced colleague can also be valuable.  (Haematology)  [RD] |  |
| 21. *Emotional debriefing:*  Allow time to process emotional impact and gain insights for future scenarios. | Human statement:  The personality of peers and senior colleagues will determine whether you can ask them for help on non-clinical issues. Be aware that issues might resurface longer-term and know who to ask for help in managing them. | Again, the personality of a senior colleague shouldn't be prejudged as to whether they can be supportive.  (Palliative Medicine)  [TM, RD]  There is probably a difference between reflection on performance and emotional debrief. Different people are likely to be better at each component (senior colleagues vs friends for instance).  (Rheumatology)  [RD, WS]  The wording of “carefully” suggests that potentially harm can come from sharing and debriefing with colleagues. Perhaps chose suitable colleagues?  (Endocrinology & Diabetes)  [RD]  I think this is tricky. Often there will be no relevant mentor within a department. The emphasis should be on promoting the development of these leadership skills in the workforce, rather than warning people off seeking support.  (Clinical Pharmacology & Therapeutics)  [TM, RD, WS] | Yes although this may be difficult for the trainee to ascertain who best to approach.  (Medical Oncology)  [RD, WS]  You should you be able to approach all seniors on non clinical issues, if not they have not been trained properly.  (Medical Oncology)  [TM, RD, WS]  Personality or previous personal interactions / experience? Not sure the ability to counsel / reflect is always personality-dependent - although I think I know what you're getting at.  (Medical Oncology)  [TM, RD, WS]  Not sure I fully understand what is meant by this.  (Medical Oncology)  [NOT CODED] | Needs a 'trustful' setting between all colleagues.  [RD, WS]  Not all colleagues care.  [RD, WS]  I don’t think this is helpful. It’s not clear what exactly is being said here or what the doctor is supposed to do with this guidance.  [TM] |
|  | AI statement:  Discussing the experience with colleagues can help process the emotional impact and gain insights for future situations. |  | This depends on the people involved.  (Haematology)  [RD, WS] | Can?  [RD, WS] |
| 22. *Team members:*  Check with junior colleagues before asking them to break bad news. | Human statement only:  If senior doctors are asking more junior staff to assist in breaking bad news, they need to check they are ok to do this in advance. | This is a core skill of any doctor, whilst it’s important to check beforehand, this shouldn't be a barrier to gaining experience.  (Palliative Medicine)  [WP, WS, SD]  Breaking bad news is an important part of training of doctors. Discussing how to do this and giving feedback to resident doctors as well as checking on their wellbeing is important.  (Geriatric Medicine)  [WP, WS]  I think this is a core part of our job, and it can occur unexpectedly. If a colleague has personal circumstances which materially impact on a key aspect of work such as this, then I think we should be promoting this being disclosed proactively via appropriate channels.  (Clinical Pharmacology & Therapeutics)  [WP, WS, DU] | Breaking bad news is something all doctors should be able to do - it is part of our role.  (Medical Oncology)  [DU, SD]  Whilst I think it is good practice to check, as someone may have a personal reason why that time is not good for them to break bad news, I think we need to be clear that breaking bad news is the responsibility of ALL healthcare professionals, no matter what stage or speciality.  (Medical Oncology)  [DU, WP]  Experience of breaking bad news if essential part of oncology training, supporting juniors through the process is important but juniors cannot be "shielded" from the experience. What is meant by in advance? Asking junior to assist in breaking bad news will depend on the situation and their level of experience and seniority at the time.  (Medical Oncology)  [DU, WP, SD]  Agree and disagree. Depends on the seniority and experience and confidence of the junior staff. I would always check with an IMT doctor particularly if limited oncology experience, but would expect my more senior trainees - ST4+ particularly - to be able to break bad news as oncology trainees.  (Medical Oncology)  [DU, WP, SD]  I find this question ambiguous, yes senior doctors should check that the junior doctor is ok to do this.  (Medical Oncology)  [WP]  It is an essential part of medical practice and it is important that more junior staff have supported experience with this as otherwise they will find it very difficult when they reach a more senior role.  (Haematology)  [WP, SD]  in general this isn’t something to randomly delegate; how to empower those in ore junior positions to decline to do this?  (Paediatric Cardiology)  [WP, WS, SD] | This is an expectation of the role and Junior staff need to observe and learn how to do this in a supported way in order to be able to manage this effectively themselves at a later stage. Exposure to this should be beneficial ultimately to helping build the skills and resilience to manage this.  [WP, SD]  But will be a beneficial learning experience.  [SD]  And rehearse this with them.  [SD]  Doing it for the first time can be hard.  [WP, SD] |
| Any other comments? | Human approach | Communication skills are complex and even with experience and training it is not always straight-forward. I think this style of question makes breaking bad news seem more a tick-box approach.  (Palliative Medicine)  [TM, SD]  I had lots of thoughts but some answered in next few questions - so the flow is a bit confusing here. Important to give doctors a structure to work to early and the reflections maybe come later?  (Palliative Medicine)  [TM, SD] | There is no right way to give bad news, the messenger is often killed. Preparation is key, knowledge gives confidence.  (Medical Oncology)  [WP, WS]  All senior staff should be available for reflection of breaking bad news.  (Medical Oncology)  [SD, RD]  Discuss the structure of the conversation… Warning shot...pause and watch response... first part of information...pause and watch patient.... further information based on initial response. Allow time for patient to process information. Understand they may stop listening and need silence. Resist the urge to keep talking to fill the silence.  (Haematology)  [TM, SD, WS]  I think the survey is full of leading questions that will invoke polarized responses and honestly, I'm not sure how helpful this survey is going to be.  (Clinical Genetics)  [TM]  I've done this as to how I might answer the Qs - the Qs however have no ambiguity in that answers are at the ends of the scale.  (Audiovestibular Medicine)  [TM] | All point above are crucial and important. Important to remember to differentiate any training, some doctors will be more able to manage and cope than others. Teaching strategies and coping mechanisms is key.  [SD, WS, RD]  Eye contact very important; take your patients seriously (no matter his / her abilities and understanding); talk to the patient without the people there with him / her.  [TM, SD, WS]  No mention of impact of bad news, e.g. may not be bad news for the doctor but is for the patient. Is it the doctors job to provide comfort?  [TM]  These statements offer insights / suggestions rather than specific 'dos or don'ts' and are a bit more open - although not all are in this format (e.g. team members: 'they need to').  [TM]  A lot of the narrative above are more statements than guidance, leaving the trainee to work out the practical application. Not sure how helpful this is- some statements leave you with more questions than answers.  [TM] |
| Any other comments? | AI approach | This version [VERSION B] seems more human and more individualised and focuses on skills rather than specific "tasks. it also aligns better with communication skills workshops.  (Palliative Medicine)  [TM, SD]  More straightforward for trainees to read and work through, but loses some of the depth/ nuance that was in version A.  (Palliative Medicine)  [TM]  Much prefer version B. Keeps approach as patient centred, gives a clear structure to approach, helpful phrases included that could be used by doctors in training.  (Palliative Medicine)  [TM, SD]  Version B is far better!!  (Palliative Medicine)  [TM]  Elements of both versions are very good. I would suggest combining the parts which are distinct between the two versions. Overall, version A seems more actionable for training.  (Respiratory Medicine)  [TM, SD]  I think version B especially (but also A to some extent) creates unrealistic expectations about what can actually be delivered in the NHS. Being able to find 5 minutes and a quiet room for this important task is usually about all that can be done, the idea that there will regularly be the possibility of written materials or referral to support workers is outlandish outside of specialist services (e.g. in oncology). The idea that nurses or consultants will have sufficient time or motivation to attend is also not realistic.  (Respiratory Medicine)  [TM]  Version B more comprehensive than version A.  (Cardiology)  [TM]  Version B is much clearer and straightforward to answer than Version A.  (Acute Internal Medicine)  [TM]  Overall I prefer Version B.  (Genitourinary Medicine)  [TM]  Focus on clearly understanding the patient and family's view prior to difficult discussions is probably the single most useful thing I was taught in medical school.  (Infectious Diseases & Tropical Medicine)  [TM, PE]  Version B is more practical and it’s better suited for a stepwise approach especially while working on a busy medical take.  (Endocrinology & Diabetes)  [TM, SD]  Includes additional information on why something is helpful or recommended.  (Geriatric Medicine)  [TM]  Version A was so poor -had it been written to deliberately inflame!  (Geriatric Medicine)  [TM] | [VERSION B] Longer with more specific examples which I think could lead to misinterpretation or use of these verbatim if seen as "best practice" which misses the point that each consultation will need slightly different tactics.  (Medical Oncology)  [TM]  I did not like B. Too clinical, prescriptive and often not relevant.  (Medical Oncology)  [TM]  There are things in B which would complement some of those in A and vice versa!  (Paediatric Cardiology)  [TM]  Version A is more general, B is more "step by step" moment by moment for those with less experience.  (Audiovestibular Medicine)  [TM, SD] | Version B is more factual but less comprehensive and well-rounded than A.  [TM]  These (Version B) statements are mainly in an imperative format (although Debrief with Colleagues isn't). I prefer the format of Version A as it allows more possibilities for contextualisation. Sometimes more tentative.  [TM]  I prefer Version B because of the clear language and the good advice given. A is a mixture of giving facts and making suggestions. Is there enough evidence for the facts and an empirical basis for the suggestions? (I suppose that is the case)  [TM]  Reassuring as a patient to know this level of thought is going into what might be seen to be really consequential in relieving distress - seems just as valuable in the mental as in the physical realm.  [TM]  if possible make sure the patient has someone with him / her - because perception is often not good enough in these situations.  [PE] |
